# Hydrocarbons in Deep-Sea Sediments following the 2010 Deepwater Horizon Blowout in the Northeast Gulf of Mexico

**DOI:** 10.1371/journal.pone.0128371

**Published:** 2015-05-28

**Authors:** Isabel C. Romero, Patrick T. Schwing, Gregg R. Brooks, Rebekka A. Larson, David W. Hastings, Greg Ellis, Ethan A. Goddard, David J. Hollander

**Affiliations:** 1 University of South Florida, College of Marine Science, St. Petersburg, Florida, 33701, United States of America; 2 Eckerd College, St. Petersburg, Florida, 33711, United States of America; University of California, Merced, UNITED STATES

## Abstract

The Deepwater Horizon (DWH) spill released 4.9 million barrels of oil into the Gulf of Mexico (GoM) over 87 days. Sediment and water sampling efforts were concentrated SW of the DWH and in coastal areas. Here we present geochemistry data from sediment cores collected in the aftermath of the DWH event from 1000 – 1500 m water depth in the DeSoto Canyon, NE of the DWH wellhead. Cores were analyzed at high-resolution (at 2 mm and 5 mm intervals) in order to evaluate the concentration, composition and input of hydrocarbons to the seafloor. Specifically, we analyzed total organic carbon (TOC), aliphatic, polycyclic aromatic hydrocarbon (PAHs), and biomarker (hopanes, steranes, diasteranes) compounds to elucidate possible sources and transport pathways for deposition of hydrocarbons. Results showed higher hydrocarbon concentrations during 2010-2011 compared to years prior to 2010. Hydrocarbon inputs in 2010-2011 were composed of a mixture of sources including terrestrial, planktonic, and weathered oil. Our results suggest that after the DWH event, both soluble and highly insoluble hydrocarbons were deposited at enhanced rates in the deep-sea. We proposed two distinct transport pathways of hydrocarbon deposition: 1) sinking of oil-particle aggregates (hydrocarbon-contaminated marine snow and/or suspended particulate material), and 2) advective transport and direct contact of the deep plume with the continental slope surface sediments between 1000-1200 m. Our findings underline the complexity of the depositional event observed in the aftermath of the DWH event in terms of multiple sources, variable concentrations, and spatial (depth-related) variability in the DeSoto Canyon, NE of the DWH wellhead.

## Introduction

Sediments serve as repository systems for a large range of organic matter sources and hydrocarbons that can be used to assess historical impacts to the environment. In the northern Gulf of Mexico (GoM), the composition of organic matter deposited in deep-sea sediments is controlled by physical sorting of particles (regional hydrodynamics) and the off-shore movement of the less dense material from terrigenous sources transported by the Mississippi River [1,2]. In shallow coastal areas (e.g. estuaries) the terrigenous pool is primarily composed of fresh vascular plant detritus whereas in deeper areas off-shore it is comprised of highly altered organic matter from angiosperm grassland soils [3,4]. Compared to sediments in shallow areas, terrigenous derived matter in deep-sea sediments is typically recalcitrant [3]. Marine-derived organic matter content in sediments is greater in areas associated with high rates of primary productivity in the surface waters [4]. Other sources of sedimentary organic carbon are erosion of sedimentary rocks from the Mississsipi River drainage basin, and fossil fuels from natural oil seeps and petroleum exploration [3,5,6].

It is estimated that an average of 95,500 tons oil enters the GoM annually from natural seeps (73%), oil and gas extraction activities (3%), transportation activities (4%), and oil combustion byproducts (~16%) [7]. In comparison, the Deepwater Horizon (DWH) spill in 2010 released 4.9 million barrels of oil into the Gulf of Mexico (~699,700 metric tons) [8], an amount over seven times the average annual input of oil into the GoM [9]. The DWH blowout was unique not only for its size but also its depth at 1500 m below the sea surface. The released oil partitioned into oil drops, gas bubbles, and gas hydrates with accompanied partitioning of petroleum hydrocarbons into aqueous, gas and particulate phases. A mixture of soluble and insoluble petroleum hydrocarbons reached the sea surface, where it evaporated (5% of the leaked mass), formed sheens and slicks (10% of the leaked mass), was mechanically recovered (20% of the leaked mass) or burned (6% of the leaked mass) [10]. In addition, a lateral plume at ~1000–1200 m depth formed (35% of the leaked mass) rich in water-soluble gases and compounds [10–14]. The final fate of the hydrocarbons from the subsurface plume is unknown, although dissolution and biodegradation have been proposed as important processes [11,14,15].

Following the blowout of the DWH drilling rig, an unusually large marine snow event was observed [16,17]. The large depositional event may have occurred by marine snow formation from surface oil slicks (containing ~0.5 million barrels of liquid oil) and the subsurface deep plume (containing ~1.7 million barrels of liquid oil). Despite the broad accounts of chemical composition of hydrocarbons at sea surface and depth in the GoM in 2010 [10] and their fate in the environment [18–28], a comprehensive discussion of possible transport pathways of oil into the deepwater sediment environment and a hydrocarbon inventory for 2010 is not available.

The primary objectives of this study are to contribute to the overall understanding of hydrocarbon geochemistry in deepwater sediments by providing information on the concentration and composition of sediments samples collected from the DeSoto Canyon, NE of the DWH; and to interpret these data within the context of the possible sources and transport pathways of hydrocarbons to the deep sea during the period of the study.

## Materials and methods

### Sample collection

**Fig 1:**
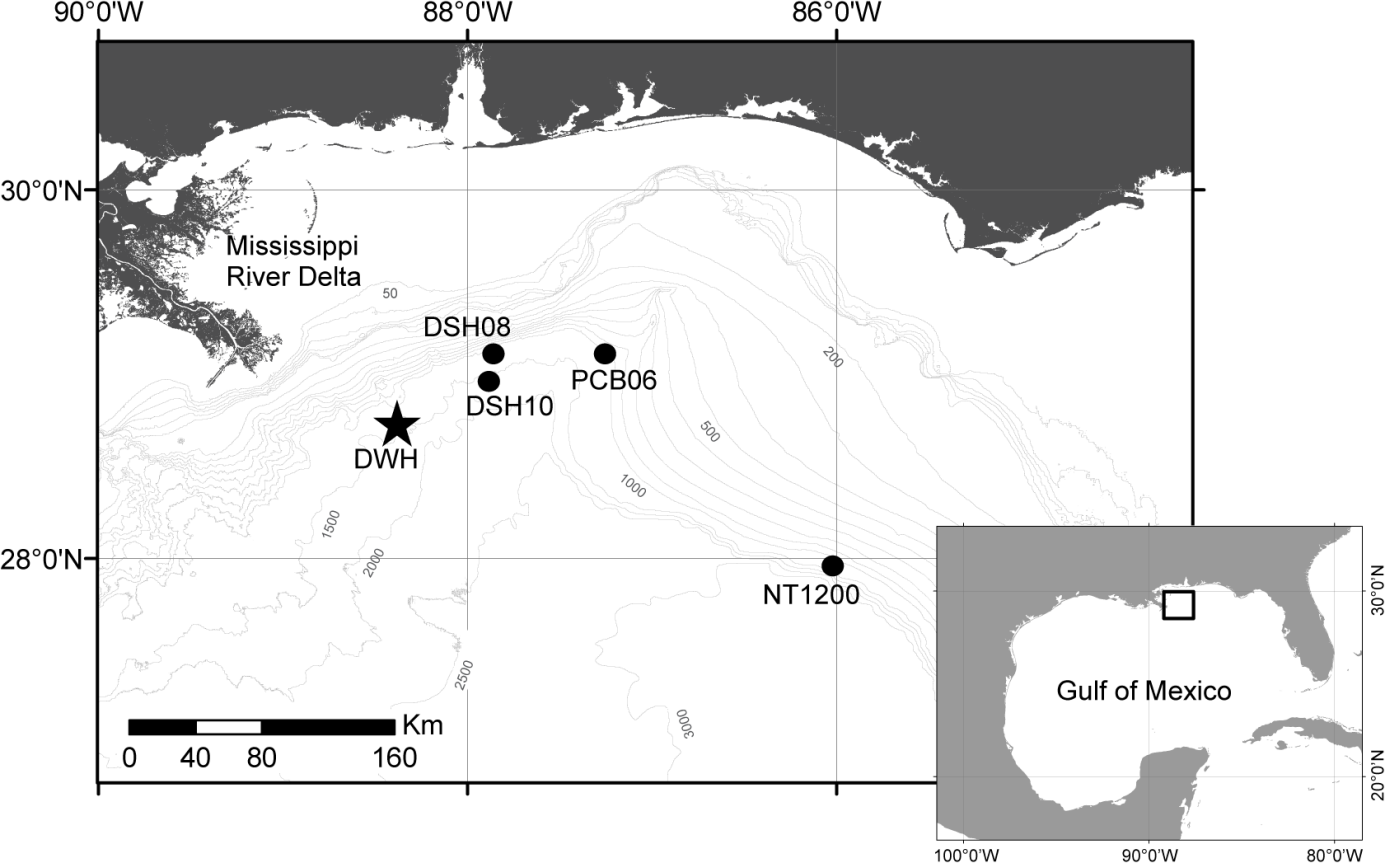
Location of study sites (DSH10, DSH08, PCB06, NT1200), Deepwater Horizon (DWH), and Mississippi River Delta. Insert show the location of the sites within the Gulf of Mexico.

Sediment cores were collected in December 2010 and February 2011 on board Florida of Institute of Oceanogrpahy’s (FIO) R/V WeatherBird II during oil spill response cruises WB1111 and WB1114. Three sediment-coring sites were located in the DeSoto Canyon in the northeastern GoM (Fig 1). Sites were located ~56 km (DSH10 at 1520 m depth, 28.59°N, 87.53°W), ~83 km (DSH08 at 1143 m depth, 29.07°N, 87.52°W) and ~111 km (PCB06 at 1043 m depth, 29.06°N, 87.16°W) northeast of the DWH. These sites were chosen based on predictions of oil transport towards the opening of the canyon due to wind direction and the formation of the Loop Current during the summer of 2010 [29,30]. Due to the lack of studies conducted before 2010 in the studied sites (DSH10, DSH08, PCB06), a core was collected 240 km east of the DWH (NT1200 at 1200 m depth, 27° 57.98’ N, 86° 1.38’ W, 1200 m depth) to provide a control site representing an area that was outside of the impact area during the DWH event.

A multi-corer (Ocean Instruments MC-800) was used to collect sediment cores with minimal disturbance to the sediment-water interface and surficial sediment intervals. Whole cores were sliced on board or in the laboratory at the University of South Florida’s College of Marine Science Paleo-Laboratory (USFCMS-PL) at 2 mm (0–20 mm) and 5 mm (>20 mm) intervals downcore. A modified version of the Engstrom [31] and Valsangkar [32] extrusion devices was used. The extruder consists of a basal metal plate holding a threaded steel rod with uniform threads calibrated (1 complete turn = 2 mm) to push the core at specific intervals vertically upward, where the sample was then sliced off the top. Extruded samples from cores designated for organic geochemistry analysis (bulk carbon and nitrogen content, stable isotopes, and hydrocarbon analyses) were stored in pre-combusted glass containers (450°C for 4hr) in a freezer (-20°C). Frozen samples were freeze-dried and ground using a mortar and pestle. Extruded samples from cores designated for analysis of short-lived radioisotopes geochronology (^210^Pb, ^234^Th) were weighed, freeze-dried, and weighed again to determine their wet mass and dry mass to calculate dry bulk density. A separate core was split and used for visual descriptions and X-ray photographs to confirm the absence of bioturbation that may compromise stratigraphy and geochronology. Sediments collected in December 2010 were analyzed down to a depth of 90 mm to include recently deposited and historical data, while sediments collected in February 2011 were analyzed down to 6 mm to analyze the most recently deposited sediment.

### Geochronology and input calculations

Short-lived radionuclide geochronology samples were counted for 48 hours on a Canberra Series HPGe (High-Purity Germanium) Coaxial Planar Photon Detectors to determine excess ^210^Pb and excess ^234^Th activities for age dating. Samples were counted within 120 days of collection to account for the short half-life (24.1 days) of ^234^Th. Raw activities were corrected for counting time, detector efficiency, as well as for the fraction of the total radioisotope measured, yielding activity in disintegrations per minute per gram (dpm/g) with error generally <5% of activity. Age dates were assigned to each sample analyzed using the Constant Rate of Supply (CRS) Model as described previously [33–35]. Organic carbon inputs to the sediment surface were calculated by combining compound concentrations with sediment density measurements and geochronology (age dates). Inputs were calculated for total organic carbon (TOC) integrated over specific 2 or 5 mm intervals corresponding to each year in the sediment cores analyzed.

### Bulk carbon, nitrogen, and stable isotopes

Bulk analyses for total organic carbon (TOC), nitrogen (N), and carbon and nitrogen isotopic values (δ^13^C, δ^15^N) were carried out at USFCMS-PL. Prior to analysis of TOC and δ^13^C, pre-weighed subsamples were placed in glass containers and acidified (80% 1.0N HCl) to remove inorganic carbon [36]. Dried subsamples were then placed in silver capsules and analyzed using a CarloeErba 2500 Series 2 Elemental Analyzer coupled to a Thermo Finnigan Delta XL. All samples were analyzed in duplicate and data reported as the average (<1% difference between duplicates). The results are reported using conventional delta notation in permil (‰) units relative to the Vienna Pee Dee Belemnite (VPDB) standard. The precision for replicate analyses of external standards (NIST 8573, NIST 8574, NIIST 1570) was 0.3‰ for δ^13^C and 0.4‰ for δ^15^N (1σ, N = 100).

### Hydrocarbon analysis

We followed modified EPA methods [37,38] and QA/QC protocols for the analysis of hydrocarbons. Samples were freeze-dried and extracted under high temperature (100°C) and pressure (1500 psi) with a solvent mixture 9:1v:v dichloromethane: methanol (MeOH) using an Accelerated Solvent Extraction system (ASE 200®, Dionex). For selected samples perdeuterated *n*-alkane (d_50_-Tetracosane) and PAHs (d_10_-acenaphthene, d_10_-phenanthrene, d_10_-fluoranthene, d_12_-benz(a)anthracene, d_12_-benzo(a)pyrene, d_14_-dibenz(ah)anthracene, d_14_-benzo(ai)perylene) were added. Activated copper (40 mesh, 99.9%, Sigma-Aldrich, USA) was added to desulfurize each sample extract. Lipid fractions were separated using solid-phase extraction (SPE) with silica/cyanopropyl glass columns (SiO_2_/C_3_-CN, 1 g/0.5 g, 6 mL) made at the USFCMS-PL. Silica gel (high purity grade, 100–200 mesh, pore size 30A, Sigma Aldrich, USA) was combusted (450°C for 4h) and deactivated (2%) previous to assemble the columns. Ultra-clean silica bonded cyanopropyl (C_3_-CN, 50 μM, Interchim, USA) was used as well to improve separation of lipid fractions. Lipid fractions were collected by sequentially eluting the extracts with hexane (100%) to collect aliphatic hydrocarbons, and hexane/dichloromethylene mixture (3:1, v:v) to collect aromatic hydrocarbons. Both fractions were concentrated and spiked with d_14_-terphenyl to correct for injection volume. All solvents used were at the highest purity available and without further purification. Two extraction blanks were included with each set of samples (15–18 samples) to ensure no contamination from chemicals, glassware and/or laboratory equipment.

The aliphatic fraction was quantified in a gas chromatograph/flame ionization detector (GC/FID) by the external standard method in splitless injections of 5μL. A VF-1ms (15m x 0.25mm x 0.25μm) capillary column was used with a GC oven temperature programming of 80°C held for 0.5 min, then increased to 320°C at a rate of 10°C min^-1^ and held for 5.5 min. Injector temperature was set to 280°C. Identification and quantification of *n*-alkanes (nC_12_-nC_40_) and isoprenoids pristine (Pr) and phytane (Phy) were conducted by comparing with reference standards. Branched alkanes and the unresolved complex mixture (UCM) were calculated using the mean response factors of *n*-alkanes. All samples were corrected for % recovery from spiked samples with surrogates and were generally 60–80%. Total aliphatic concentration was calculated as the sum of *n*-alkanes, isoprenoids, branched alkanes and UCM. Aliphatic compounds are expressed as sediment dry weight concentrations.

The aromatic fraction was quantified in a Gas Chromatograph/mass spectrometric detector (GC/MS) in full scan mode (*m/z* 50–550). Splitless injections of 1μL of the sample were conducted. A RXi®5sil column was used with a GC oven temperature programming of 60°C held for 8 min, then increased to 290°C at a rate of 6°C/min and held for 4 min, then increased to 340°C at a rate of 14°C/min, and held at the upper temperature for 5 min. The temperature of the MS detector was 250°C. Target PAH (polycyclic aromatic hydrocarbon) compounds are 2-ring: Naphthalene (N) and alkylated homologues (N_C1-C4_), 3-ring: Acenaphthylene (ACL), Acenaphthene (ACE), Fluorene (F), Dibenzothiophene (D), Phenanthrene (P), Anthracene (AN), and their alkylated homologues (P/AN_C1-C4_, D_C1-C2_), 4-ring: Fluoranthene (FL), Pyrene (PY), Benz[a]anthracene (BAA), Chrysene (C), and their alkylated homologues (FL/PY_C1-C4_, BAA/C_C1-C4_), 5-ring: Benzo[b]fluoranthene (BBF), Benzo[k]fluoranthene (BKF), Benzo[a]pyrene (BAP), Dibenz[a,h]anthracene (DA), and alkylated homologues (BP/PER_C1-C4_), and 6-ring: Indeno[1,2,3-cd]pyrene (ID), Benzo[ghi]perylene (BGP). Concentrations of PAHs were calculated using response factors by comparison with a known standard mixture (16-unsubstituited EPA Priority Pollutants and selected congeners: Ultrascientific US-106N PAH mix, NIST 1491a) and were corrected for the recovery of the surrogate standard. When no commercial reference standard was available, compounds were quantitated using the response factor for an isomer. Therefore, the concentrations determined for many of the alkylated PAHs were semiquantitative. Recoveries from spiked samples included with each batch were generally within QA/QC criteria of 60–120%. Aromatic compounds are expressed as sediment dry weight concentrations.

Biomarkers (hopanes and steranes) were quantified using GC/MS/MS multiple reaction monitoring (MRM) on a Varian 320 triple quadrupole MS. Splitless injections of 1μL of the sample were conducted. Chromatographic separation of biomarker compounds were conducted using a RXi®5sil column (30 m x 0.25 mm x 0.25 μm) with a GC oven temperature programming of 80°C held for 1 min, then increased to 200°C at a rate of 40°C/min, to 250°C at 5°C/min, to 300°C at 2°C/min, to 320°C at 10°C/min, and held for 2 min. The GC was operated in constant-flow mode (1ml/min) with an inlet temperature of 275°C and a transfer line temperature of 320°C. Ion source temperature was 180°C and source electron energy was 70eV. Mass transitions targeted appropriate parent molecular ion masses on Q1 and monitored mass 191.2 (hopanes) or 217.0 (steranes) on Q3, with collision energy held at 10 volts throughout. Targeted hopanes (and relevant Q1 masses) included C_27_, C_28_, and C_29_ norhopanes (370.5, 384.5, and 398.5, respectively), C_30_ hopanes (412.5), and C_31_ through C_35_ homohopanes (426.5, 440.5, 454.5, 468.5, and 482.5), while sterane targets included C_27_- C_29_ steranes and diasteranes (372.7, 386.7, and 400.7). An additional transition (376.7 to 221) was monitored to quantify the internal standard (cholestane 2,2,4,4 D4; CDN Isotopes). Argon at a pressure of 1 millitorr was used as a collision gas. Concentrations of biomarkers compounds were calculated using response factors by comparison with a known standard mixture (Hopane/Sterane calibration mix, Chiron, S-4436-10-IO) and the internal standard. When no commercial reference standard was available, compounds were quantitated using the response factor for the nearest available homologue in the same compound class. Concentrations were corrected for the recovery of the surrogate standard (d_50_-Tetracosane) for the F1 fraction. Recoveries from spiked samples included with each batch were generally within QA/QC criteria of 60–80%. Biomarker compounds are expressed as sediment dry weight concentrations.

Replicate hydrocarbon analyses were done in selected samples from the cores collected in 2010. Our depth resolution of the core samples allowed us to replicate hydrocarbon analyses at the 65–85 mm depth interval. The relative standard deviations (RSDs) of replicates (N = 4) for PAHs analysis were 10%, 17% and 19% for DSH10, DSH08 and PCB06 sites, respectively. RSDs of replicates (N = 4) for aliphatic analysis were 12%, 6% and 10% for DSH10, DSH08 and PCB06 sites, respectively. And, RSDs of replicates (N = 4) for biomarker analysis were 17%, 22% and 4% for DSH10, DSH08 and PCB06 sites, respectively. RSDs values for all compound groups were lower than the variability observed in concentration from surface sediment layers to downcore in all sites (see Results section).

Diagnostic ratios were calculated to discriminate hydrocarbon sources in the samples collected. Diagnostic ratios use isomer pairs that are abundant in different PAH sources but with similar dissolution and adsorption properties as they have comparable thermodynamic partitioning and kinetic mass transfer coefficients [39]. Typically, low molecular weight PAHs (LMW, containing 2–3 ring PAHs) are abundant in petrogenic sources while high molecular weight PAHs (HMW, containing 4–6 ring PAHs) are abundant in pyrogenic sources. However, some oils like the DWH oil contain HMW PAHs, but in moderate levels relative to LMW PAHs [14]. HMW PAHs can become more abundant due to loss of LMW PAHs during weathering processes (e.g. dissolution), therefore PAH diagnostic ratios must be interpreted with caution. We used the following diagnostic ratios: AN/(P+AN), pyrogenic index, PI: ∑(other 3–6 ring EPA priority pollutant PAHs) / ∑(5 alkylated PAHs), HMW/LMW, Parental/alkyl, (BAP+BGP)/HMW, and (ID+C)/HMW [40–43]. We also calculated diagnostic ratios using biomarkers due to their properties of source specificity and resistance to weathering and biodegradation [44–46]. We used the following diagnostic ratios: Ts/Tm (18α(H)-22,29,30-trisnorneohopane/17α(H)-22,29,30-trisnorhopane), C29ββ(S+R)/C29αα(S+R) (24-ethyl-5α(H),14α(H),17α(H)-20(S+R)-cholestane/24-ethyl-5α(H),14β(H),17β(H)-20(S+R)-cholestane), C29ααS/H (24-ethyl-5α(H)14α(H)17α(H)-20S-cholestane/17α(H)21β(H)-hopane).

For comparison to potential hydrocarbon sources, diagnostic ratios were calculated for DWH crude oil (Macondo oil, MC252 block) obtained from British Petroleum (BP, sample No. SOB-20100622-084) and from crude oil standard reference material collected from the insertion tube that was receiving oil directly from the Macondo well (NIST 2779). We also used analyses from two sediment samples collected at the DWH wellhead that contain high concentration of 17α(H)21β(H)-hopane (60 ng g^-1^). In addition, we used GoM sediment for PAH ratios from sites with evidence of pyrogenic (HMW) PAH inputs reported by the Operational Science Advisory Team in 2010 [47].

## Results

### Bulk measurements

**Fig 2:**
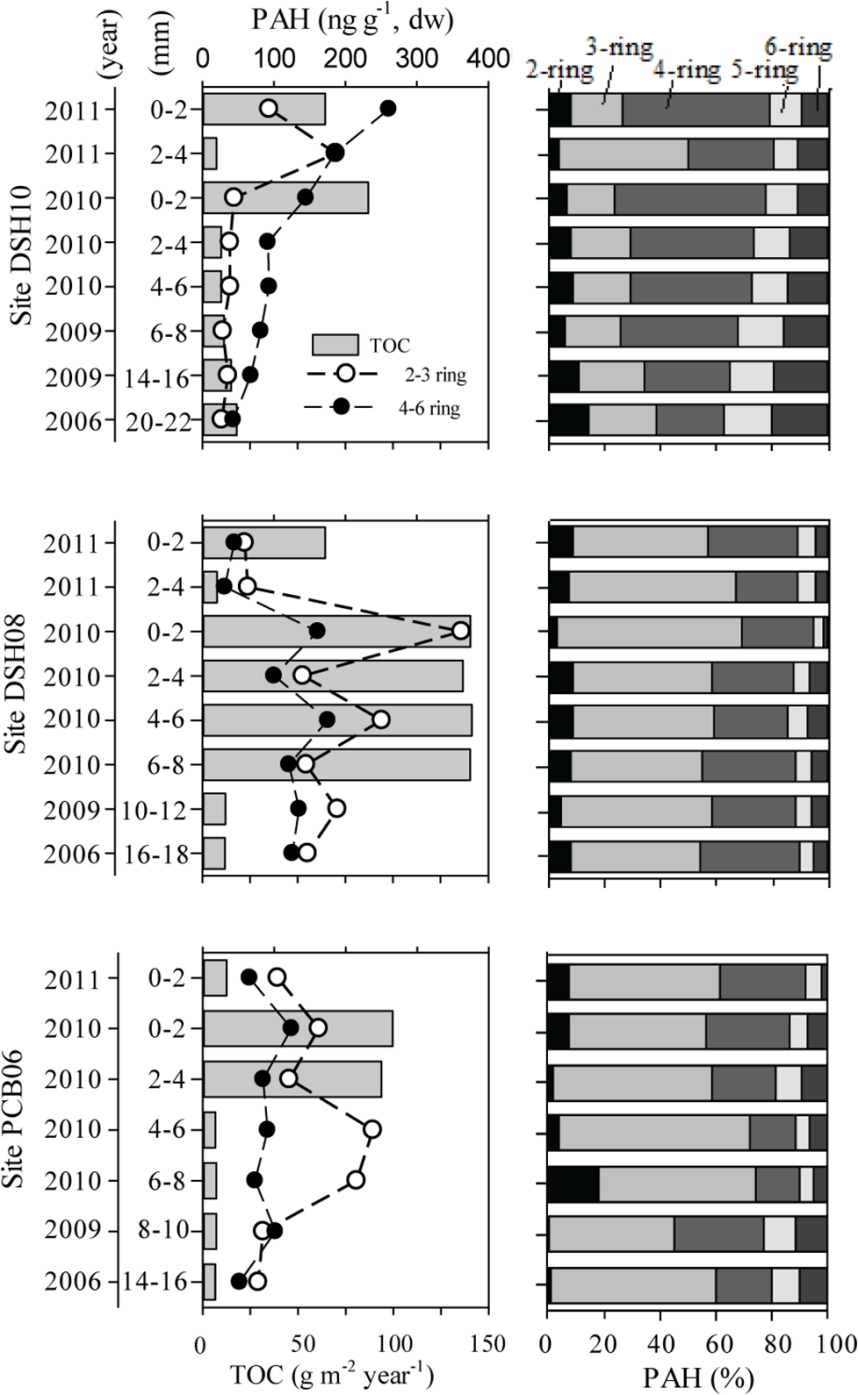
TOC rates, 2–3 ring (low molecular weight, LMW) and 4–6 ring (high molecular weight, HMW) PAH concentrations, and PAH composition profiles (2-, 3-, 4-, 5-, 6-ring) for each study site (DSH10, DSH08, PCB06). Data shown are for cores collected in 2010 (interval: 2010 to 2006) and 2011 (only 2011 data). 2-ring: Naphthalene and alkylated homologues; 3-ring: Acenaphthylene, Acenaphthene, Fluorene, Dibenzothiophene, Phenanthrene, Anthracene, and alkylated homologues; 4-ring: Fluoranthene, Pyrene, Benz[a]anthracene, Chrysene, and alkylated homologues; 5-ring: Benzo[b]fluoranthene, Benzo[k]fluoranthene, Benzo[a]pyrene, Dibenz[a,h]anthracene, and alkylated homologues; 6-ring: Indeno[1,2,3-cd]pyrene, Benzo[ghi]perylene.

In the DeSoto Canyon, recently deposited sediment sections were established in each sedimentary record indicating variable and larger deposition in 2010 compared to previous years (Table 1, S1 Table). TOC input was from 2 to 4 times higher in 2010 compared to previous years (Fig 2), indicating that in 2010 TOC content in surface sediments is primarily the result of variation in water column-derived organic matter input.

**Table 1:** Aliphatic ratios and concentrations for the sediment cores collected in 2010 (interval: 2010 to ~1970) and 2011 (only 2011 data) at the study sites.

Site	Year	Depth Interval (mm)	TOC (%)	δ^13^C (‰)	δ^15^N (‰)	*n*-alkanes							UCM (μg g^-1^)
						C_17_/Pr	C_18_/Phy	Total CPI_14-35_	CPI_14-23_	CPI_26-35_	C≤25 (μg g^-1^)	C ≥26 (μg g^-1^)	
**DSH10**	2011	0–2	1.8	-20.3	6.1	3.8	1.0	2.6	1.8	2.7	0.2	4.4	28.5
	2011	2–4	1.8	-23.1	6.0	n.d.	0.8	2.4	2.6	2.3	0.1	1.8	65.9
	***Mean***	***0–4***	***1*.*8***	***-21*.*7***	***6*.*0***		***0*.*9***	***2*.*5***	***2*.*2***	***2*.*5***	***0*.*2***	***3*.*1***	***47*.*2***
	2010	0–2	1.3	-20.6	5.5	0.5	0.0	2.4	1.4	2.9	0.3	1.4	41.3
	2010	2–4	1.2	-20.5	5.3	0.2	1.2	3.4	2.4	3.6	0.3	2.6	11.9
	2010	4–6	1.2	-20.6	5.3	0.5	1.4	3.8	2.3	4.1	0.3	3.3	8.5
	***Mean***	***0–6***	***1*.*2***	***-20*.*6***	***5*.*4***	***0*.*4***	***0*.*9***	***3*.*2***	***2*.*0***	***3*.*6***	***0*.*3***	***2*.*5***	***20*.*6***
	***CI***	*** ***	***(0*.*1)***	***(0*.*1)***	***(0*.*1)***	***(0*.*2)***	***(0*.*9)***	***(0*.*8)***	***(0*.*6)***	***(0*.*7)***	***(0*.*1)***	***(1*.*1)***	***(20*.*4)***
	2009	6–8	1.2	-20.6	5.1	0.1	1.4	3.4	2.6	3.6	0.2	2.6	5.8
	2009	8–10	1.2	-20.5	5.1	0.0	n.d.	0.6	0.8	0.5	0.4	1.3	3.5
	2008	10–12	1.2	-20.7	5.0	0.3	1.1	4.3	3.0	4.6	0.2	2.4	6.0
	2007	12–14	1.3	-20.9	5.0	0.0	4.3	3.1	2.1	3.3	0.2	1.6	6.7
	2007	14–16	1.3	-20.9	4.4	0.1	0.5	2.3	1.4	2.5	0.3	2.0	6.4
	2006	16–18	1.4	-21.0	4.7	0.2	1.5	4.5	2.8	4.9	0.0	0.4	8.9
	2006	18–20	1.5	-20.8	4.7	0.2	0.7	4.6	2.8	5.0	0.2	2.0	8.1
	2005	20–22	1.5	-20.9	4.7	0.9	1.0	4.6	2.6	5.1	0.2	1.3	4.8
	2005	22–24	1.4	-21.0	4.6	0.7	1.7	4.4	2.6	4.8	0.2	3.0	6.0
	2004	24–26	1.3	-20.9	4.5	0.0	n.d.	5.8	2.5	7.2	0.0	0.3	7.4
	2004	26–28	1.3	-20.9	4.6	1.5	1.1	4.6	2.9	4.9	0.3	3.5	4.3
	2003	28–30	1.4	-20.9	4.5	0.9	1.7	4.5	2.7	4.9	0.2	2.2	3.8
	2003	30–35	1.5	-21.0	4.4	1.5	1.5	1.2	2.4	0.9	1.1	5.1	0.2
	2001	35–40	1.4	-20.8	4.4	0.7	0.5	3.2	3.1	3.2	0.2	1.9	5.5
	1999	40–45	1.2	-20.3	4.4	1.3	1.3	3.0	2.5	3.1	0.9	7.7	0.0
	1996	50–55	1.4	-20.4	4.6	1.5	1.3	0.8	1.7	0.1	1.4	2.0	3.2
	1994	55–60	1.4	-20.5	4.4	1.1	0.4	3.6	3.2	3.7	0.2	1.5	5.2
	1992	60–65	1.4	n.d.	4.4	1.3	1.4	0.8	2.3	0.3	0.9	2.6	9.4
	1976	85–90	1.4	n.d.	4.4	1.3	1.4	2.0	2.3	1.9	1.1	3.9	0.0
	***Mean***	***6–90***	***1*.*4***	***-20*.*8***	***4*.*6***	***0*.*7***	***1*.*3***	***3*.*0***	***2*.*4***	***3*.*4***	***0*.*4***	***2*.*5***	***5*.*0***
	***CI***	*** ***	***(<0*.*1)***	***(0*.*1)***	***(0*.*1)***	***(0*.*3)***	***(0*.*4)***	***(0*.*6)***	***(0*.*3)***	***(0*.*9)***	***(0*.*2)***	***(0*.*8)***	***(1*.*3)***
**DHS08**	2011	0–2	1.9	-21.1	6.1	3.8	1.0	4.4	2.6	4.7	0.3	3.7	41.1
	2011	2–4	1.6	-20.7	6.0	n.d.	0.8	4.1	2.4	4.4	0.3	2.2	12.8
	***Mean***	***0–4***	***1*.*8***	***-20*.*9***	***6*.*0***	*** ***	***0*.*9***	***4*.*3***	***2*.*5***	***4*.*6***	***0*.*3***	***3*.*0***	***27*.*0***
	2010	0–2	2.0	-20.1	5.5	1.0	1.7	1.3	1.9	1.3	0.4	5.1	325.7
	2010	2–4	1.9	-20.4	5.4	0.5	1.9	2.5	1.0	3.4	0.7	3.5	48.0
	2010	4–6	2.0	-22.5	5.7	n.d.	3.1	3.0	1.1	4.1	0.2	1.3	73.9
	2010	6–8	2.0	-19.1	5.6	0.7	1.8	2.6	1.2	3.2	0.7	4.5	41.1
	2010	8–10	2.0	-20.7	5.6	0.5	1.5	2.8	1.2	3.8	0.9	3.3	36.8
	***Mean***	***0–10***	***2*.*0***	***-20*.*5***	***5*.*6***	***0*.*7***	***2*.*0***	***2*.*5***	***1*.*3***	***4*.*1***	***0*.*6***	***3*.*6***	***105*.*1***
	***CI***	*** ***	***(<0*.*1)***	***(1*.*1)***	***(0*.*1)***	***(0*.*2)***	***(0*.*6)***	***(0*.*6)***	***(0*.*3)***	***(1*.*0)***	***(0*.*2)***	***(1*.*3)***	***(108*.*8)***
	2009	10–12	2.0	-20.4	5.5	1.1	1.7	2.5	*1*.*2*	3.5	1.4	4.5	42.9
	2008	12–14	2.0	-20.6	5.4	0.8	1.5	2.9	*1*.*2*	3.7	1.0	5.9	42.8
	2007	14–16	2.0	-20.3	5.5	0.8	2.1	3.0	*1*.*3*	3.6	0.7	5.5	43.9
	2006	16–18	2.0	-20.7	5.4	0.4	30.0	2.9	*0*.*9*	4.9	0.6	2.2	54.5
	2005	18–20	1.9	-20.6	5.4	0.6	1.7	2.9	*0*.*8*	4.5	0.6	2.7	43.5
	2003	20–25	1.9	-20.7	5.2	0.9	1.5	3.5	1.7	4.0	1.3	11.4	17.9
	2000	25–30	2.0	-20.6	5.1	0.6	2.0	3.1	1.3	3.8	0.6	3.5	31.5
	1996	30–35	1.8	-20.8	5.2	1.0	1.8	3.1	1.5	3.7	1.4	10.0	13.3
	1993	35–40	1.9	-20.7	5.2	0.0	2.6	3.3	1.5	3.7	0.9	10.9	16.7
	1990	40–45	1.9	-20.7	4.9	0.0	3.2	3.5	1.8	3.9	0.6	8.7	15.1
	1985	45–50	1.8	-20.9	5.0	0.0	2.9	3.5	1.9	3.9	0.8	10.0	11.4
	1983	50–55	1.8	-20.9	5.0	0.0	3.3	3.6	*1*.*6*	4.3	0.7	7.5	12.4
	1979	55–60	1.8	-20.8	5.1	1.2	2.1	3.3	*1*.*4*	4.2	1.0	6.5	7.0
	***Mean***	***10–60***	***1*.*9***	***-20*.*7***	***5*.*2***	***0*.*6***	***4*.*4***	***3*.*2***	***1*.*4***	***4*.*0***	***0*.*9***	***6*.*9***	***27*.*1***
	***CI***	*** ***	***(<0*.*1)***	***(0*.*1)***	***(0*.*1)***	***(0*.*2)***	***(4*.*2)***	***(0*.*2)***	***(0*.*2)***	***(0*.*2)***	***(0*.*2)***	***(1*.*7)***	***(8*.*9)***
**PCB06**	2011	0–2	1.9	-20.9	6.1	0.0	3.4	1.9	1.2	1.9	0.3	0.4	24.4
	2010	0–2	1.3	-20.5	4.5	n.d.	1.7	3.0	1.3	3.5	0.2	1.4	33.4
	2010	2–4	1.2	-20.6	4.0	2.0	1.7	3.8	1.8	4.5	0.2	1.3	20.6
	2010	4–6	1.2	-20.5	4.2	1.1	1.3	2.5	1.4	2.8	0.2	1.7	27.3
	2010	6–8	1.2	-20.4	4.3	n.d.	n.d.	2.4	1.4	2.7	0.1	1.1	18.9
	***Mean***	***0–8***	***1*.*2***	***-20*.*5***	***4*.*2***	***1*.*5***	***1*.*6***	***2*.*9***	***1*.*5***	***3*.*4***	***0*.*2***	***0*.*2***	***25*.*1***
	***CI***	*** ***	***(<0*.*1)***	***(0*.*1)***	***(0*.*2)***	*** ***	***(1*.*1)***	***(0*.*5)***	***(0*.*2)***	***(0*.*7)***	***(0*.*1)***	***(0*.*2)***	***(6*.*5)***
	2009	8–10	1.2	-20.5	4.3	0.3	2.9	4.0	1.8	4.7	0.3	3.3	13.2
	2008	10–12	1.2	-20.6	4.4	0.6	1.6	2.7	1.6	3.1	0.4	2.6	17.6
	2006	12–14	1.3	-20.6	4.1	0.1	2.9	2.9	1.7	3.1	0.3	3.4	15.9
	2005	14–16	1.3	-20.3	4.1	0.4	1.7	2.6	1.9	2.7	0.3	3.5	17.2
	2004	16–18	1.4	-20.7	4.2	n.d.	1.3	4.3	2.0	5.0	0.3	3.4	12.3
	2002	18–20	1.5	-20.7	3.8	n.d.	1.1	3.0	1.7	3.3	0.2	2.2	14.8
	2001	20–22	1.5	-20.5	5.1	n.d.	1.8	3.3	1.5	4.1	0.5	3.3	9.6
	2000	22–24	1.4	-20.5	5.1	n.d.	2.0	2.2	1.1	2.7	0.3	1.5	9.2
	1998	24–26	1.3	-20.3	4.7	n.d.	2.3	3.4	1.7	4.0	0.2	2.0	8.8
	1997	26–28	1.3	-20.5	4.9	n.d.	1.8	3.5	1.5	4.5	0.1	0.9	7.5
	1996	28–30	1.4	-20.4	4.9	n.d.	n.d.	2.6	1.5	2.9	0.2	1.8	12.3
	1993	30–35	1.5	-20.5	4.8	n.d.	1.2	3.8	2.0	4.3	0.6	5.6	2.4
	1989	36–38	1.4	-20.5	4.6	n.d.	1.3	3.6	2.1	4.0	0.6	6.0	0.8
	1986	40–45	1.7	-20.6	4.2	n.d.	1.5	3.9	2.0	4.4	0.4	4.6	2.3
	1982	45–50	1.5	-20.3	4.4	2.6	3.3	2.6	1.7	2.8	0.3	3.5	2.0
	1978	50–55	1.3	-20.2	4.5	n.d.	1.2	4.6	2.0	5.5	0.4	4.5	0.9
	1974	55–60	0.9	-20.1	4.2	1.4	3.5	3.2	1.1	4.5	1.3	7.4	0.0
	1970	60–65	1.4	-20.4	n.d.	n.d.	1.9	3.0	1.5	3.6	0.4	2.3	2.3
	***Mean***	***8–65***	***1*.*7***	***-20*.*5***	***4*.*5***	***0*.*9***	***2*.*0***	***3*.*3***	***1*.*7***	***3*.*9***	***0*.*4***	***3*.*4***	***8*.*3***
	***CI***	*** ***	***(0*.*1)***	***(0*.*1)***	***(0*.*2)***	***(0*.*4)***	***(0*.*4)***	***(0*.*3)***	***(0*.*3)***	***(0*.*9)***	***(0*.*1)***	***(0*.*8)***	***(2*.*9)***
**NT1200**	***Mean***	***0–22***	***n*.*d*.**	***n*.*d*.**	***n*.*d*.**	***0*.*9***	***n*.*d*.**	***3*.*0***	***1*.*7***	***3*.*8***	***0*.*2***	***1*.*8***	***n*.*d*.**
** **	***CI***	*** ***	*** ***	*** ***	*** ***	***(0*.*3)***	*** ***	***(0*.*4)***	***(1*.*5)***	***(0*.*8)***	***(0*.*1)***	***(0*.*4)***	*** ***
**DWH oil**	***Mean***	*** ***	***n*.*d*.**	***-27*.*4***	***n*.*d*.**	***1*.*6***	***2*.*3***	***0*.*9***	***1*.*6***	***0*.*9***	***45*.*0***	***9*.*9***	***n*.*d*.**
** **	***CI***	*** ***	*** ***	*** ***	*** ***	***(0*.*1)***	***(0*.*2)***	***(<0*.*1)***	***(<0*.*1)***	***(<0*.*1)***	***(2*.*2)***	***(0*.*8)***	*** ***

Averages shown as arithmetic mean ± CI. CPI (Carbon preference index) = ∑ odd Cn / ∑ even Cn, for each specific range of *n*-alkanes.

**Fig 3:**
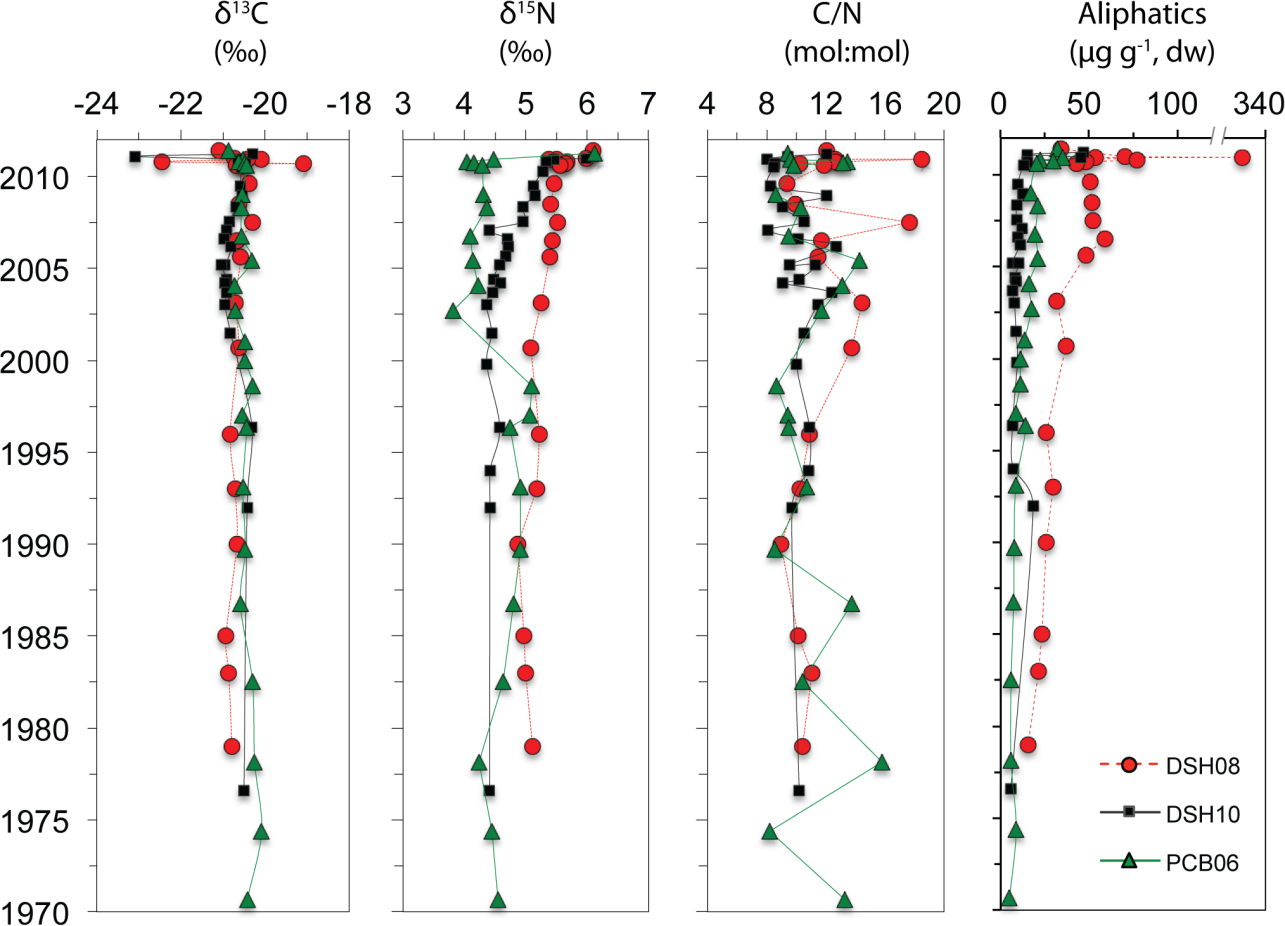
Geochemical profiles for cores collected in 2010 and 2011 at the study sites (DSH08, DSH10, PCB06). Downcore depth intervals for each year are in Table 2.

The δ^13^C of organic carbon in the sediment samples showed a higher variation in 2010 at DSH08 (from -19‰ to -23‰), and in 2011 at DSH10 (-20‰ to -23‰) than in previous years (Fig 3, Table 1). δ^15^N values were similar down core in all sites (5.4 ± 0.1‰ at DSH10, 5.6 ± 0.1‰ at DSH08, and 4.2 ± 0.1‰ at PCB06) but higher in 2011 (up to ~6.1‰ at all sites, Fig 3). C:N is higher in 2010 at DSH08 and PCB06, and in 2011 at DSH10 compared to previous years, although downcore C:N ratios showed large variations for all sites (Fig 3). The general trends observed in the bulk measurements indicate higher variation of organic carbon sources and inputs in 2010 at DSH08 and PCB06, and 2011 at DSH10 compared to previous years.

### Aliphatics in sediments

Total concentration of aliphatics in the DeSoto Canyon varied from 5 to 337 μg g^-1^. Higher concentrations were observed in 2010 for shallower sites (up to 337 and 35 μg g^-1^ for DSH08 and PCB06, respectively; Fig 3) and in 2011 for the deeper site (up to 46 μg g^-1^ for DSH10; Fig 3). There was a sharp increase in aliphatic concentration in most sediment intervals corresponding to 2010 (in DSH08 and PCB06) and 2011 (in DSH10 and DSH08). The intermittent increase in concentration observed in the surface sediments is indicative of a recent sedimentary depositional event (Fig 1). This is supported by the trend observed in UCM (unresolved complex mixtures of hydrocarbons) (Table 1). Elevated concentrations of UCM are generally attributed to petroleum hydrocarbons in contaminated sediments [48–50]. The presence of UCM was observed in all sites in nearly all years, but higher concentrations of at least one order of magnitude were found in some sediment intervals during 2010 (Table 1). Moreover, there were still high concentrations of UCM in 2011 for two sites (DSH10 and PCB06).

Long-chain (C ≥ 26) and short-chain (C ≤ 25) *n*-alkanes varied in concentration (Table 1). In a consistent pattern, long-chain *n*-alkanes accounted for >80% of the total *n*-alkanes in all years for all sites indicating dominance of terrestrial inputs. Similarly, calculations of the value of CPI_14-35_ indicated a predominance of odd-numbered carbon chains in all years (Table 1) typical of compounds from terrestrial vascular plants [50–52].

Isoprenoids (Pr and Phy) are more resistant to degradation than *n*-alkanes resulting in a decrease of the ratios C_17_/Pr and C_18_/Phy in weathered samples [52–54]. Lower C_17_/Pr and C_18_/Phy ratios were observed in more recently deposited sediments (corresponding to 2010) than in downcore sediments, probably due to a rapid deposition of weathered hydrocarbons (Table 1). In 2011, isoprenoid ratios changed for all sites suggesting a different source of organic matter. In some 2011 samples high ratios are present (>2) indicating a more biogenic input to the surface sediments. In downcore sediment layers corresponding to 2006, high values were observed in C_18_/Phy and low values in C_17_/Pr for the shallower sites (DSH08 and PCB06) suggesting a high deposition of biogenic material post Hurricane Katrina.

The general trends observed in the aliphatic fraction indicate a mixture of organic carbon sources and increased concentrations in 2010 (DSH08 and PCB06) and 2011 (DSH10) in contrast to previous years. In contrast, similar organic carbon sources and low concentrations were observed in all depth intervals (2011 to 1990) in the control site, NT1200 (Table 1, S1 Fig). Aliphatic ratios in the control site are similar to data from prior years to 2010 observed in the other study sites (Table 1).

### PAHs in sediments

Total PAH concentration in the DeSoto Canyon varied from 70 to 524 ng g^-1^. Higher concentrations were observed in 2010 for the shallower sites (up to 524 and 329 ng g^-1^ for DSH08 and PCB06, respectively) and in 2011 only for the deeper site (up to 373 ng g^-1^ for DSH10) (Table 2, Fig 2). Also, a disparity between TOC input and PAH concentrations of LMW and HMW compounds was observed in 2010 and 2011 (Fig 2). For PCB06, LMW PAHs were higher at the 4–8 mm interval followed by an increase of TOC flux at the 0–4 mm interval. For DSH08, LMW PAHs showed two peaks (the 0–2 mm and 4–6 mm intervals) concurrent with a high TOC flux (0–8 mm). In contrast, LMW PAHs at DSH10 show an increase in 2011 at the 2–4 mm interval when TOC flux is low. DSH10 was the only location with higher HMW than LMW PAH concentrations and the HMW PAHs consistently increased from the end of 2010 to 2011 when TOC flux was high (Fig 2). In contrast, for the control site (NT1200) LMW and HMW PAHs were consistently low in all depth intervals (Table 2, S1 Fig).

**Table 2:** Concentration of PAH compounds in sediment samples from cores collected in 2010 (interval: 2010 to ~2002) and 2011 (only 2011 data) at the studied sites (units: ng g^-1^) and DWH oil (units: μg g^-1^).

Site	Year	Sed. Depth (mm)	PAHs																							
			N	ACL	ACE	F	D	P	AN	FL	PY	BAA	C	BBF	BKF	BAP	ID	DA	BGP	N (C1-C4)	P/AN (C1-C4)	FL/PY (C1-C4)	BAA/C (C1-C4)	BP/PER (C1-C4)	D (C1-C2)	Total PAH
**DSH10**	2011	0–2	0.0	0.8	0.0	2.0	0.0	17.5	1.6	14.7	10.9	17.4	15.8	26.7	0.0	0.1	15.4	0.0	18.3	29.2	41.0	14.2	113.3	14.3	1.6	354.7
	2011	2–4	0.0	1.4	0.0	2.7	1.4	31.3	79.3	13.1	8.7	13.7	14.6	19.9	0.0	9.7	19.9	0.0	21.8	13.3	55.4	6.5	55.1	2.8	2.0	372.8
	***Mean***	***0–2***	***0*.*0***	***1*.*1***	***0*.*0***	***2*.*3***	***0*.*7***	***24*.*4***	***40*.*4***	***13*.*9***	***9*.*8***	***15*.*6***	***15*.*2***	***23*.*3***	***0*.*0***	***4*.*9***	***17*.*6***	***0*.*0***	***20*.*1***	***21*.*3***	***48*.*2***	***10*.*3***	***84*.*2***	***8*.*6***	***1*.*8***	***363*.*8***
	2010	0–2	1.0	0.6	0.0	0.7	0.7	7.8	0.8	6.5	7.5	8.2	7.3	14.8	0.0	4.0	10.1	0.0	10.9	11.1	22.1	7.0	66.4	2.8	0.0	190.2
	2010	2–4	0.0	0.7	0.0	0.7	0.0	7.5	0.9	5.9	7.1	6.9	7.4	13.6	0.0	3.0	9.5	0.0	8.7	10.7	18.2	3.4	26.2	0.5	0.0	130.9
	2010	4–6	1.0	0.6	0.0	0.5	0.6	7.7	0.9	0.0	8.0	6.5	6.0	14.4	0.0	3.3	10.4	0.0	8.6	10.4	17.3	5.4	30.9	0.0	0.0	132.6
	***Mean***	***0–6***	***0*.*7***	***0*.*7***	***0*.*0***	***0*.*6***	***0*.*4***	***7*.*7***	***0*.*9***	***4*.*1***	***7*.*5***	***7*.*2***	***6*.*9***	***14*.*3***	***0*.*0***	***3*.*4***	***10*.*0***	***0*.*0***	***9*.*4***	***10*.*7***	***19*.*2***	***5*.*2***	***41*.*1***	***1*.*1***	***0*.*0***	***151*.*2***
	***CI***	*** ***	***(0*.*7)***	***(0*.*1)***	***(0*.*0)***	***(0*.*1)***	***(0*.*4)***	***(0*.*2)***	***(0*.*1)***	***(4*.*1)***	***(0*.*6)***	***(1*.*0)***	***(0*.*9)***	***(0*.*7)***	***(0*.*0)***	***(0*.*6)***	***(0*.*5)***	***(0*.*0)***	***(1*.*4)***	***(0*.*4)***	***(2*.*9)***	***(2*.*1)***	***(24*.*9)***	***(1*.*7)***	***(0*.*0)***	***(38*.*2)***
	2009	6–8	0.0	0.2	0.0	0.4	0.4	4.6	0.6	0.1	6.2	6.5	6.0	14.2	0.0	2.6	9.7	0.0	7.9	6.5	16.0	3.2	24.3	1.3	0.0	110.6
	2007	14–16	0.0	0.9	0.0	0.2	0.5	6.9	0.8	5.0	7.4	5.0	5.0	12.9	0.0	3.2	10.5	0.0	10.2	11.2	15.5	1.6	7.4	0.0	0.0	104.0
	2004	24–26	2.8	0.4	0.0	0.2	0.4	2.8	0.6	3.4	5.1	3.3	3.4	9.3	0.0	2.4	8.1	0.0	6.2	7.2	12.8	1.5	0.6	0.3	0.0	70.6
	***Mean***	***6–26***	***1*.*4***	***0*.*6***	***0*.*0***	***0*.*2***	***0*.*4***	***4*.*9***	***0*.*7***	***4*.*2***	***6*.*2***	***4*.*1***	***4*.*2***	***11*.*1***	***0*.*0***	***2*.*8***	***9*.*3***	***0*.*0***	***8*.*2***	***9*.*2***	***14*.*1***	***1*.*5***	***4*.*0***	***0*.*1***	***0*.*0***	***87*.*3***
	***CI***	*** ***	***(1*.*9)***	***(0*.*4)***	***(0*.*0)***	***(0*.*2)***	***(0*.*1)***	***(2*.*3)***	***(0*.*1)***	***(2*.*8)***	***(1*.*3)***	***(1*.*8)***	***(1*.*5)***	***(2*.*9)***	***(0*.*0)***	***(0*.*5)***	***(1*.*4)***	***(0*.*0)***	***(2*.*3)***	***(2*.*8)***	***(2*.*0)***	***(1*.*1)***	***(13*.*8)***	***(0*.*8)***	***(0*.*0)***	***(24*.*3)***
**DSH08**	2011	0–2	0.0	0.2	0.0	1.2	0.2	4.6	0.5	3.6	5.2	1.3	4.9	5.2	0.0	0.9	1.9	0.0	2.7	9.1	38.4	7.7	10.8	0.8	0.0	99.3
	2011	2–4	0.0	0.3	0.0	0.9	0.0	7.4	0.5	3.6	4.4	0.4	3.6	4.1	0.0	1.3	1.8	0.0	3.1	7.1	47.7	2.5	5.8	0.6	0.0	95.2
	***Mean***	***0–4***	***0*.*0***	***0*.*3***	***0*.*0***	***1*.*1***	***0*.*1***	***6*.*0***	***0*.*5***	***3*.*6***	***4*.*8***	***0*.*8***	***4*.*2***	***4*.*6***	***0*.*0***	***1*.*1***	***1*.*9***	***0*.*0***	***2*.*9***	***8*.*1***	***43*.*0***	***5*.*1***	***8*.*3***	***0*.*7***	***0*.*0***	***97*.*2***
	2010	0–2	0.0	0.8	0.0	1.8	1.0	14.2	2.1	12.8	22.4	3.1	20.4	0.0	9.2	4.8	6.2	1.1	5.7	15.3	326.0	10.6	62.6	2.6	1.4	524.3
	2010	2–4	0.0	0.8	0.0	2.5	1.0	17.5	1.1	0.0	34.2	0.0	9.2	12.1	1.7	0.0	7.7	1.2	8.5	20.4	91.1	12.1	13.7	0.0	0.0	234.9
	2010	4–6	0.0	1.1	0.0	6.2	0.0	37.4	0.0	0.0	56.4	7.4	17.2	18.2	0.0	10.9	15.5	0.0	18.5	36.6	169.8	14.5	17.1	0.0	0.0	426.8
	2010	6–8	0.0	0.8	0.0	2.1	0.0	17.4	1.2	12.8	29.2	3.1	10.7	10.3	0.0	3.6	8.4	1.2	8.5	17.6	102.6	7.5	26.0	0.0	1.2	264.1
	***Mean***	***0–8***	***0*.*0***	***0*.*9***	***0*.*0***	***3*.*2***	***0*.*5***	***21*.*6***	***1*.*1***	***6*.*4***	***35*.*6***	***3*.*4***	***14*.*4***	***10*.*1***	***2*.*7***	***4*.*8***	***9*.*5***	***0*.*9***	***10*.*3***	***22*.*5***	***172*.*4***	***11*.*2***	***29*.*9***	***0*.*7***	***0*.*6***	***362*.*5***
	***CI***	*** ***	***(0*.*0)***	***(0*.*2)***	***(0*.*0)***	***(2*.*3)***	***(0*.*6)***	***(12*.*0)***	***(1*.*0)***	***(8*.*4)***	***(16*.*7)***	***(3*.*4)***	***(6*.*0)***	***(8*.*6)***	***(5*.*0)***	***(5*.*2)***	***(4*.*7)***	***(0*.*7)***	***(6*.*4)***	***(10*.*9)***	***(122*.*4)***	***(3*.*3)***	***(25*.*4)***	***(1*.*5)***	***(0*.*8)***	***(155*.*0)***
	2009	10–12	0.0	1.3	0.0	4.4	0.0	15.2	2.7	21.9	6.1	5.5	14.2	10.2	0.0	4.6	10.2	2.0	9.7	14.5	147.8	21.6	28.2	1.3	2.2	323.6
	2006	16–18	0.0	0.7	0.0	3.1	1.1	23.1	6.3	15.3	41.0	3.7	8.2	9.0	0.0	3.3	6.8	1.5	7.5	21.1	91.0	9.1	19.6	1.0	1.0	273.5
	***Mean***	***10–18***	***0*.*0***	***1*.*0***	***0*.*0***	***3*.*8***	***0*.*6***	***19*.*2***	***4*.*5***	***18*.*6***	***23*.*6***	***4*.*6***	***11*.*2***	***9*.*6***	***0*.*0***	***3*.*9***	***8*.*5***	***1*.*7***	***8*.*6***	***17*.*8***	***119*.*4***	***15*.*4***	***23*.*9***	***1*.*1***	***1*.*6***	***298*.*5***
**PCB06**	2011	0–2	0.0	0.3	0.0	1.0	0.0	6.1	0.0	0.0	5.2	1.3	5.4	4.9	0.0	1.9	0.0	0.1	4.2	13.0	84.8	10.4	29.6	2.6	0.0	170.7
	2010	0–2	0.0	0.8	0.0	1.0	0.7	12.6	0.8	8.8	11.2	2.4	8.1	11.6	2.0	3.8	10.4	0.0	9.7	20.9	125.0	33.2	22.0	1.1	1.0	287.0
	2010	2–4	0.0	0.2	0.0	0.0	0.5	5.4	0.4	5.1	8.3	1.6	6.9	11.4	2.3	2.6	11.4	1.4	8.4	3.1	110.6	7.1	17.1	1.0	1.1	206.0
	2010	4–6	0.0	0.2	0.0	1.1	1.0	22.7	0.9	9.0	11.0	1.9	7.9	12.0	1.8	2.5	9.8	0.0	10.7	12.7	154.5	7.1	15.2	2.0	0.8	285.0
	2010	6–8	0.0	0.0	0.0	0.8	0.6	6.6	0.3	4.9	11.0	1.3	6.1	9.5	1.8	1.1	7.1	0.0	8.0	52.4	153.1	7.8	14.0	0.9	1.6	288.9
	***Mean***	***0–8***	***0*.*0***	***0*.*3***	***0*.*0***	***0*.*7***	***0*.*7***	***11*.*8***	***0*.*6***	***7*.*0***	***10*.*4***	***1*.*8***	***7*.*3***	***11*.*1***	***2*.*0***	***2*.*5***	***9*.*7***	***0*.*4***	***9*.*2***	***22*.*3***	***135*.*8***	***13*.*8***	***17*.*1***	***1*.*3***	***1*.*1***	***266*.*7***
	***CI***	*** ***	***(0*.*0)***	***(0*.*4)***	***(0*.*0)***	***(0*.*6)***	***(0*.*2)***	***(9*.*0)***	***(0*.*3)***	***(2*.*6)***	***(1*.*6)***	***(0*.*5)***	***(1*.*1)***	***(1*.*2)***	***(0*.*3)***	***(1*.*3)***	***(2*.*1)***	***(0*.*8)***	***(1*.*4)***	***(24*.*1)***	***(24*.*4)***	***(14*.*6)***	***(4*.*0)***	***(0*.*6)***	***(0*.*4)***	***(45*.*9)***
	2009	8–10	0.0	0.3	0.0	0.2	0.0	6.8	0.7	7.2	13.4	2.6	7.6	12.1	2.4	3.7	10.4	1.6	11.1	0.1	69.9	10.1	18.7	1.0	0.9	180.8
	2002	14–16	0.0	0.1	0.0	0.1	0.1	6.2	0.3	4.2	4.4	1.4	4.3	8.1	1.2	2.0	6.8	0.0	6.0	1.6	63.8	3.6	7.8	2.0	0.1	124.1
	***Mean***	***8–16***	***0*.*0***	***0*.*2***	***0*.*0***	***0*.*2***	***0*.*1***	***6*.*5***	***0*.*5***	***5*.*7***	***8*.*9***	***2*.*0***	***5*.*9***	***10*.*1***	***1*.*8***	***2*.*8***	***8*.*6***	***0*.*8***	***8*.*5***	***0*.*9***	***66*.*9***	***6*.*9***	***13*.*2***	***1*.*5***	***0*.*5***	***152*.*5***
**NT1200**	***Mean***	***0–22***	***1*.*6***	***0*.*7***	***0*.*2***	***0*.*1***	***0*.*0***	***9*.*4***	***0*.*2***	***0*.*9***	***0*.*2***	***0*.*3***	***0*.*2***	***1*.*5***	***0*.*2***	***0*.*5***	***1*.*0***	***0*.*0***	***1*.*0***	***4*.*7***	***4*.*7***	***0*.*3***	***0*.*5***	***0*.*1***	***0*.*0***	***28*.*2***
	***CI***	*** ***	***(1*.*5)***	***(0*.*6)***	***(0*.*5)***	***(0*.*2)***		***(6*.*1)***	***(0*.*3)***	***(0*.*5)***	***(0*.*3)***	***(0*.*3)***	***(0*.*2)***	***(1*.*6)***	***(0*.*4)***	***(0*.*9)***	***(1*.*0)***		***(1*.*0)***	***(2*.*6)***	***(3*.*0)***	***(0*.*3)***	***(0*.*3)***	***(0*.*3)***	*** ***	***(7*.*4)***
**DWH oil**	***Mean***	*** ***	848.3	8.6	14.9	159.4	59.8	327.5	11.7	4.4	17.6	7.0	54.5	6.5	0.0	2.3	0.0	2.2	2.1	7001.3	2267.2	403.1	430.7	0.0	0.0	11629

Averages shown as arithmetic mean ± CI.

The intermittent increase in concentration observed only in the surface of all sediment cores except NT1200, indicates a recent sedimentary depositional event in the DeSoto Canyon. This is supported by the notion that PAHs are known to be relatively persistent in sediments due to their hydrophobicity and particle adsorption affinity [55]. PAH preservation in the sedimentary environment occurs on a decadal time scale [56,57] while LMW PAHs have a half-life of days (e.g. anthracene) [56]. In our study, we observed some LMW PAH compounds with higher levels downcore in the years between 2004 to 2009 (Table 2) suggesting that degradation of these compounds may be low at these sites.

Additionally, the relative composition of PAHs by their number of rings showed a larger variation in 2010 and 2011 than in previous years. DSH10 showed a distinct distribution of PAHs in 2011 (~15% increase in 4-rings, up to 25% increase in 3-rings, ~10% decrease in 6-rings; Fig 2) with diagnostic ratios suggesting a mixed source of pyrogenic (abundant HMW PAHs) and petrogenic PAHs (Table 3). Also, there were differences in the relative composition of PAHs at DSH08 in 2010 and 2011 (~16% increase in 3-rings, ~10% decrease in 4-rings; Fig 2), but PAHs present were primarily from a petrogenic origin (Table 3). There was also a distinct variation in the composition of PAHs at PCB06 in 2010 (up to ~20% increase in 2-rings, ~10% increase in 3-rings, and ~15% decrease in 4-rings; Fig 2) also primarily composed of PAHs from a petrogenic origin (Table 3).

**Table 3:** PAH diagnostic ratios for the cores collected in 2010 (interval: 2010 to ~2002) and 2011 (only 2011 data) at the studied sites, DWH oil, and a sediment sample (GoM sed.) reported by OSAT 1 (2010).

Site	Year	Sed. Depth (mm)	PI	An/(Phe+An)	HMW/LMW	Parental/Alkyl	(ID+C)/HMW	(BAP+BGP)/HMW
**DSH10**	2011	0–2	0.42	0.08	2.8	0.7	0.12	0.07
	2011	2–4	1.01	0.72	1.0	1.7	0.19	0.17
	***Mean***	***0–4***	***0*.*72***	***0*.*40***	***1*.*9***	***1*.*2***	***0*.*15***	***0*.*12***
	2010	0–2	0.50	0.10	3.2	0.7	0.12	0.10
	2010	2–4	0.76	0.11	2.4	1.2	0.18	0.13
	2010	4–6	0.66	0.10	2.4	1.1	0.18	0.13
	***Mean***	***0–6***	***0*.*64***	***0*.*10***	***2*.*7***	***1*.*0***	***0*.*16***	***0*.*12***
	***CI***	*** ***	***(0*.*15)***	***(0*.*01)***	***(0*.*6)***	***(0*.*3)***	***(0*.*04)***	***(0*.*02)***
	2009	6–8	0.77	0.12	2.9	1.1	0.19	0.13
	2007	14–16	1.16	0.10	1.9	1.9	0.23	0.20
	2004	24–26	1.21	0.18	1.6	2.1	0.26	0.20
	***Mean***	***6–26***	***1*.*05***	***0*.*13***	***2*.*1***	***1*.*7***	***0*.*23***	***0*.*17***
	***CI***		***(0*.*28)***	***(0*.*04)***	***(0*.*8)***	***(0*.*6)***	***(0*.*04)***	***(0*.*05)***
**DHS08**	2011	0–2	0.28	0.09	0.8	0.5	0.15	0.08
	2011	2–4	0.26	0.06	0.5	0.5	0.17	0.14
	***Mean***	***0–4***	***0*.*27***	***0*.*08***	***0*.*7***	***0*.*5***	***0*.*16***	***0*.*11***
	2010	0–2	0.15	0.13	0.4	0.3	0.16	0.07
	2010	2–4	0.40	0.06	0.7	0.7	0.17	0.08
	2010	4–6	0.43	0.00	0.7	0.8	0.19	0.17
	2010	6–8	0.43	0.07	0.8	0.7	0.16	0.10
	***Mean***	***0–8***	***0*.*35***	***0*.*06***	***0*.*7***	***0*.*6***	***0*.*17***	***0*.*10***
	***CI***		***(0*.*13)***	***(0*.*05)***	***(0*.*2)***	***(0*.*2)***	***(0*.*01)***	***(0*.*04)***
	2009	10–12	0.30	0.15	0.7	0.5	0.18	0.11
	2006	16–18	0.53	0.21	0.9	0.9	0.12	0.09
	***Mean***	***10–18***	***0*.*42***	***0*.*18***	***0*.*8***	***0*.*7***	***0*.*15***	***0*.*10***
**PCB06**	2011	0–2	0.12	0.00	0.6	0.2	0.08	0.09
	2010	0–2	0.27	0.06	0.8	0.4	0.15	0.11
	2010	2–4	0.35	0.07	0.7	0.5	0.22	0.13
	2010	4–6	0.27	0.04	0.5	0.5	0.20	0.15
	2010	6–8	0.18	0.04	0.3	0.3	0.18	0.12
	***Mean***	***0–8***	***0*.*27***	***0*.*05***	***0*.*6***	***0*.*40***	***0*.*18***	***0*.*13***
	***CI***		***(0*.*07)***	***(0*.*02)***	***(0*.*2)***	***(0*.*1)***	***(0*.*03)***	***(0*.*02)***
	2009	8–10	0.57	0.09	1.3	0.8	0.18	0.14
	2002	14–16	0.38	0.05	0.7	0.6	0.21	0.16
	***Mean***	***8–16***	***0*.*48***	***0*.*07***	***1*.*0***	***0*.*7***	***0*.*20***	***0*.*15***
**NT1200**	***Mean***	***0–22***	***0*.*30***	***0*.*03***	***0*.*3***	***2*.*2***	***0*.*25***	***0*.*13***
	***CI***	*** ***	***(0*.*16)***	***(0*.*05)***	***(0*.*2)***	***(1*.*1)***	***(0*.*23)***	***(0*.*12)***
References	DWH oil	*** ***	0.01	0.03	0.1	0.2	0.06	0.00
	GoM sed.^(1)^	*** ***	3.92	0.28	4.0	4230	0.15	0.17
	Petrogenic source	*** ***	<0.30	<0.10	<1.0	<1.0	<0.15	<0.17
	Pyrogenic source	*** ***	>0.30	>0.10	>1.0	>1.0	>0.15	>0.17

Averages shown as arithmetic mean ± CI.

(1) GoM sediment data from sites with evidence of pyrogenic input collected in 2010 (OSAT 1, 2010).

### Hopanes in sediments

**Fig 4:**
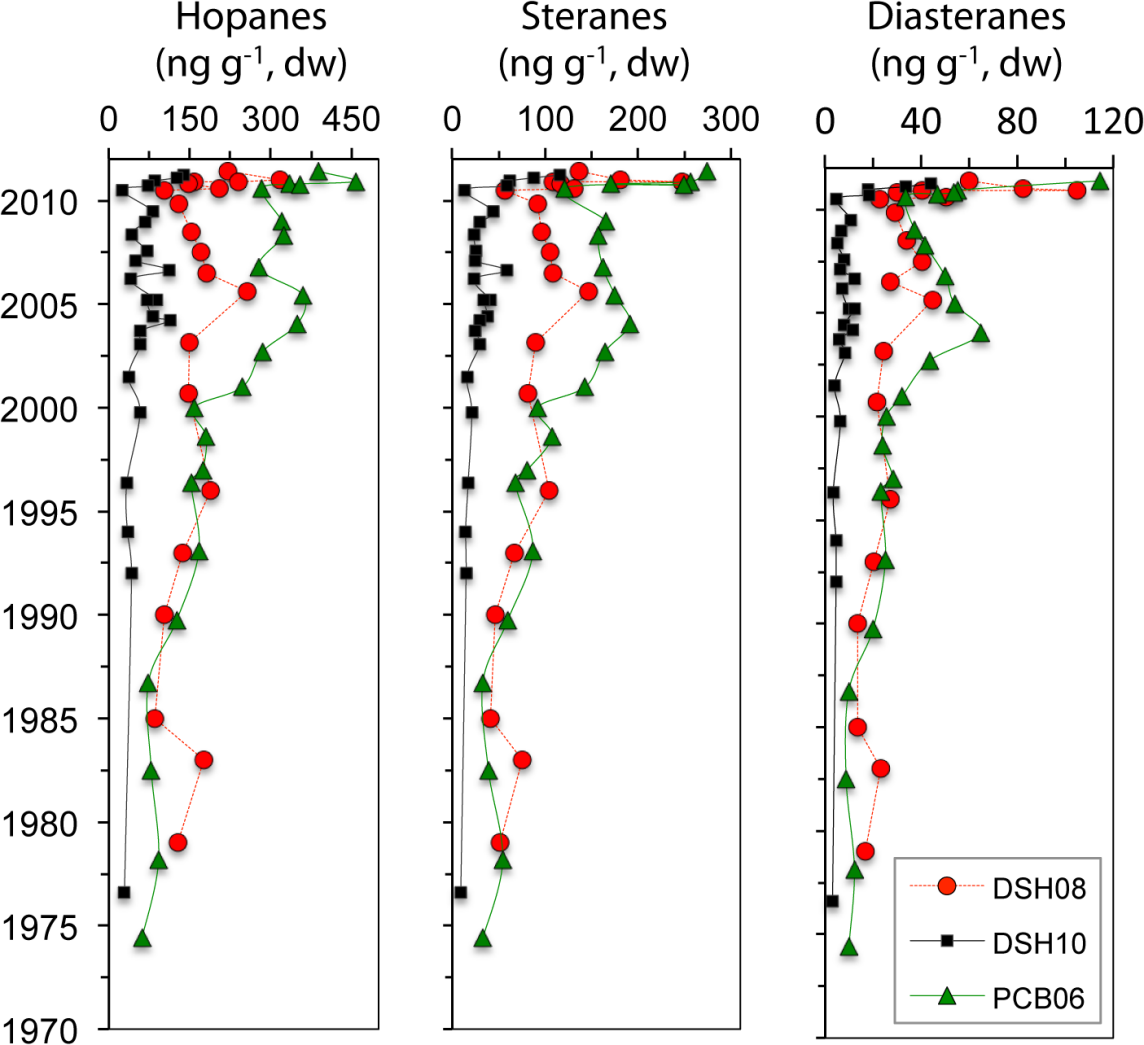
Biomarker profiles for the cores collected in 2010 and 2011 at the studied sites (DSH10, DSH08, PCB06).

Total concentration of biomarkers in the DeSoto Canyon varied from 41 to 776 ng g^-1^ (Fig 4). Higher concentrations of hopanes (up to 317 and 457 ng g^-1^ for DSH08 and PCB06, respectively), steranes (up to 247 and 274 ng g^-1^ for DSH08 and PCB06, respectively), and diasteranes compounds (up to 105 and 115 ng g^-1^ for DSH08 and PCB06, respectively) were observed in 2010–2011 for the shallower sites. Higher concentrations of biomarkers at the deeper site (DSH10) were observed only in 2011 (up to 139, 116 and 44 ng g^-1^ for hopanes, steranes and diasteranes, respectively). In contrast, for the control site (NT1200) biomarkers were consistently low in all depth intervals (up to 60, 20.6 and 5.2 ng g^-1^ for hopanes, steranes and diasteranes, respectively; S1 Fig). Higher concentrations were only observed in the surface intervals of all sediment cores except NT1200, indicating a recent sedimentary depositional event in the DeSoto Canyon, as suggested as well by aliphatic and PAH compounds (Fig 1). A less pronounced peak was observed downcore (at ~2006) in the shallower sites in the DeSoto Canyon indicating a depositional event post Hurricane Katrina.

Elevated concentrations of biomarkers are generally attributed to petroleum hydrocarbons in contaminated sediments due to their recalcitrant nature and source-specific compound distribution. The distribution profiles of biomarkers in our samples and in GoM oils are dominated by 17α(H), 21β(H)-hopane compound. In addition, C27–C29 diasteranes compounds are abundant with the 20R isomers more abundant than the 20S isomers. Typical biomarker ratios used in forensic analysis have shown to be useful in identifying the DWH event as the source-oil in samples collected from beaches (sand, rocks, tar balls) [45,46]. However, some biomarker compounds can degrade compromising their use to some extent for source oil identification [45,52]. We found diagnostic ratios of known recalcitrant biomarkers (Table 4) to vary between crude oil (MC252 oil, NIST standard) and contaminated sediment samples (see the method section for details). Vertical transport of spilled oil through the 1.5 km water column and horizontal transport to the study sites weathered biomarker compounds, as observed in a deep-water coral community [28]. Thus, biomarker ratios from deep-sea samples should be interpreted with caution. Most ratios determined for 2010–2011 samples lie within the values of MC252 oil and contaminated sediment samples (with ±20% analytical uncertainty), indicating a match with DWH oil (Table 4). Specifically, DSH10 showed a match with MC252 oil, DSH08 matched both with MC252 oil and contaminated sediments, and PCB06 mostly matched MC252 oil. In contrast, most ratios determined for pre-2010 samples did not match MC252 oil or the contaminated sediment samples, indicative of other sources for biomarkers. Similarly, the control site (NT1200) did not match MC252 oil or the contaminated sediment samples in all depth intervals (Table 4).

**Table 4:** Biomarker diagnostic ratios for the cores collected in 2010 (interval: 2010 to ~2002) and 2011 (only 2011 data) at the studied sites.

Site	Year	Sed. Depth (mm)	n	Ts/Tm	C29ααS/H	C29ββ(S+R)/C29αα(S+R)
**DSH10**	2011	0–4	2	1.06	0.47	1.2
	2010	0–6	3	1.14	0.46	1.15
				-0.46	-0.04	-0.22
	Pre-2010	Jun-90	19	0.77	0.27	1.22
				-0.21	-0.05	-0.19
**DHS08**	2011	0–4	2	0.87	0.29	1.63
	2010	0–8	5	0.78	0.34	1.34
				-0.18	-0.06	-0.21
	Pre-2010	Oct-60	19	0.73	0.28	1.3
				-0.07	-0.04	-0.12
**PCB06**	2011	0–2	1	0.39	0.65	1.16
	2010	0–8	4	1.1	0.39	1.32
				-0.77	-0.04	-0.34
	Pre-2010	Aug-65	18	0.55	0.31	1.16
				-0.09	-0.03	-0.15
**NT1200**	2011–1990	0–22	7	0.61	1.58	0.25
				-0.11	-0.3	-0.07
References	NIST^(1)^		3	1.15	0.4	1.38
				-0.22	-0.03	-0.15
	MC252^(2)^		3	1.29	0.58	1.37
				-0.19	-0.22	-0.35
	DWH sed^(3)^		1	1.14	0.45	1.14
	DWH sed^(4)^		1	0.81	0.37	1.07

Averages shown as arithmetic mean ± CI.

(1)Reference oil: NIST 2779

(2)Reference oil: British Petroleum (sample No. SOB-20100622-084)

(3)Contaminated sediment sample collected at DWH wellhead

(4)Contaminated sediment sample collected at DWH wellhead.

## Discussion

### Hydrocarbon concentrations

The results from the sites in the DeSoto Canyon indicate that higher amounts of hydrocarbons reached deepwater sediments in the study area in 2010–2011 for the shallower sites (DSH08 and PCB06) and in 2011 for the deeper site (DSH10) than in previous years. Specifically, aliphatic, PAH and biomarkers concentrations were higher in 2010–2011 (up to 337 μg g^-1^, 525 ng g^-1^, and 776 ng g^-1^, respectively) compared to previous years (up to 50 μg g^-1^, 320 ng g^-1^, and 523 ng g^-1^respectively) and the control site (up to 30.4 μg g^-1^, 45.0 ng g^-1^, and 85.9 ng g^-1^respectively) (Figs 2, 3 and 4; S1 Fig). Comparison with previous studies using similar analytical methods and target PAHs (parent and alkylated homologues) indicates surface sediments in the DeSoto Canyon during 2000–2002 contain lower levels of PAHs (114 ± 47 ng g^-1^) [58] relative to the elevated concentrations we observed in 2010 and 2011. Also, elevated concentrations in 2010–2011 are close to historical data from shallow water sites adjacent to and in the Mississippi River Delta (PAHs concentration decade 2000: ~600 ng g^-1^, decade 1980: ~800 ng g^-1^) [59,60] but lower than urbanized and industrialized areas worldwide (only parental PAH concentration: ~1200–11000 ng g^-1^) [61,62].

Overall, the levels of hydrocarbons reported in our study for 2010–2011 can be classified as low to moderately polluted based on the proposed range of potential impact of 10–100 μg g^-1^ for aliphatics and 100–1100 ng g^-1^ for PAHs [63,64]. Also, the observed PAH concentrations in our study area are lower than the “low limit” of PAH concentration ranges (ERL) associated with biologically adverse effects in shallow sediments [65]. However, calculation of PAH inputs using total PAH concentrations and TOC fluxes (e.g. [66]) shows high inputs of toxic hydrocarbons in 2010 (DSH08: 9816 μg m^-2^ y^-1^, PCB06: 4032 μg m^-2^ y^-1^) and 2011 (DSH10: 1368 μg m^-2^ y^-1^) compared to previous years (DSH08: 132–146 μg m^-2^ y^-1^, PCB06: 242–338 μg m^-2^ y^-1^, DSH10: 66–95 μg m^-2^ y^-1^) suggesting a potential ecological risk to deep-water environments. Fluxes in the DeSoto Canyon from 2010 are higher than previously observed in surface sediments close to the Mississippi River for PAHs (2500 μg m^-2^ y^-1^) [60] and TOC (0.9–54 g m^-2^ y^-1^) [1].

### Hydrocarbon sources

Organic matter in the continental shelf sediments of the GoM is derived from marine primary productivity, terrestrial and aged soil from watershed runoff from the Atchafalaya and Mississippi Rivers, and coastal plants debris and resuspended sediments [4,67,68]. Most of the suspended particulate material is deposited within 30 km off the Mississippi River and a gradient of terrestrial vs. marine sources is observed towards deeper environments [68]. At ~200 km from the Mississippi River delta in the DeSoto Canyon terrestrial inputs continue to dominate [69] as observed in the sediment cores from our study sites with *n*-alkanes typical of terrestrial sources (Table 1). Transport and deposition of pyrogenic PAHs also occur along with sediment deposition near the Mississippi River mouth and transported elsewhere in the GoM [68,70], as observed downcore in our samples (1970–2009) with HMW PAH concentrations higher or similar to LMW PAH concentrations.

Petroleum derived hydrocarbons in GoM sediments are consistently present and are suggested to originate from natural seeps, gas hydrate deposits, and oil exploration [6,71–74]. Natural seeps can account for most of the annual input of oil in the GoM (~73%) [7], and their significant presence in the environment can be detected when depleted stable carbon isotope values are observed in sediments (-24.1‰) [75]. Downcore samples prior to 2010 in our study (1970–2009) indicate sedimentary δ^13^C values of -20.6 ± 0.1‰, typical of a mixed organic carbon source including terrestrial-derived organic carbon from the Mississippi river drainage basin found in deep water sediments [67,76]. Previous studies indicate as well absence of seeps at specific sites in the DeSoto Canyon [58]. We conclude that during the period covered in our study (1970–2011) concentration and composition of hydrocarbon compounds at our study sites were not influenced directly by hydrocarbon inputs from natural seeps.

Petroleum derived hydrocarbons, distinct from DWH oil, were observed downcore (pre-2010) in all sites indicating the constant presence of oil at lower levels that enters the GoM annually through natural and anthropogenic activities [7]. Recalcitrant compounds such as biomarkers are useful for estimating background conditions in the GoM sedimentary environment. Mean background concentrations were 13 ± 3 ng g^-1^ for 17α(H)21β(H)-hopane, and 96 ± 18 ng g^-1^ for total biomarkers (N = 19) at ~1500 m depth, and 40 ± 7 ng g^-1^ for 17α(H)21β(H)-hopane, and 301 ± 46 ng g^-1^ for total biomarkers (N = 37) at 1000–1200 m depth in the DeSoto Canyon. Depth variability rather than location in the DeSoto Canyon has a larger influence on the magnitude and time of hydrocarbon deposition during the period covered in our study. Similarly, depth-related gradients of deposited organic matter have been observed elsewhere in the northern GoM [1,3].

Elevated concentrations of aliphatic, PAH and biomarker compounds were observed in most sediment depth intervals corresponding to the period 2010–2011. These compound groups indicate a mixture of hydrocarbons sources deposited during 2010–2011 that includes terrestrial, planktonic and weathered oil. Long-chain *n*-alkanes distribution suggests predominance of terrestrial sources, while higher concentrations of UCM, PAHs, and biomarkers indicate oil-derived hydrocarbons (Figs 2, 3 and 4). Furthermore, diagnostic ratios of biomarker compounds suggest DWH oil reached the seafloor in the DeSoto Canyon during 2010–2011 (Table 4). However, highly weathered deposited hydrocarbons as indicated by the isoprenoids ratios (Table 1), complicate the use of diagnostic ratios for source oil identification. Spilled oil from the DWH wellhead underwent weathering during both lateral and vertical transport primarily from partitioning of the more soluble compounds into the water column [10–12,14] and enhanced microbial activity [16,17,77]. Nevertheless, the time series data revealed elevated concentrations of hydrocarbons (from more degradable to recalcitrant compounds) at specific intervals in the surface of the seafloor, indicating a depositional event at all sites in the DeSoto Canyon during 2010–2011, post DWH event.

### Transport pathways of hydrocarbons

Two mechanisms of hydrocarbon deposition to the seafloor have been proposed: a sedimentary depositional pulse driven by the formation and rapid settling of contaminated particles (“flocculent blizzard” hypothesis), and the direct contact of the deep plume with the continental slope surface sediments at depths between 1000–1200 m (“bathtub ring” hypothesis). In our study, we found several different lines of geochemical evidence that support each of these hypotheses and explain how hydrocarbons reached the sedimentary environment in the DeSoto Canyon during 2010–2011.

Aggregation of suspended particulate material with dispersed crude oil and dissolved hydrocarbons in the water column promotes transport of spilled oil in the environment and toxicity in sediments. This phenomenon of oil-mineral interaction and sedimentation has been studied and documented since the 1970’s in the Exxon Valdez and Arabian Gulf oil spills and in laboratory experiments [78,79]. It is believed that oil-mineral aggregates are insignificant in locations far from coastal areas due to minimal suspended particulate material concentrations [78]. However, in 2010 the increased release of water from the Mississippi River with nutrients and fine-grain clays coupled with the long trajectory of DWH oil to the sea surface, and the abundance of oil in the water column (~5.5x10^6^ kg of hydrocarbons at the deep plume) [10] may have enhanced oil-mineral aggregation and enhanced the observed depositional event. Additionally, the increase in microbial activity after the DWH blowout [20,77] stimulated the formation of a large marine snow event in the GoM that also may have promoted the formation of oil-mineral aggregates [16,17]. This explains the presence of flocculent material on top of deep corals [28], impact on benthic communities [27], and hydrocarbon inputs to the seafloor (this study).

The observed marine snow during the summer of 2010 in the GoM consisted of phytoplankton cells, bacteria, and oil-particle aggregates [16]. Our sediment analyses from the Desoto Canyon indicate deposition of oil-particle aggregates (-23.1‰ at DSH10 in 2011, and -22.5‰ at DSH08 in 2010), biogenic material (abundant *n*-alkanes C≥25 in all sites, dominance of marine inputs at DSH08 in 2010 with δ^13^C-TOC = -19.1‰), and degraded oil and organic matter (all sites) at specific depth intervals corresponding to 2010–2011. Chronological comparison (before and after the DWH event) of source inputs using aliphatics composition indicates little variation, therefore high inputs of background algal and terrestrial biomass in 2010–2011. As hypothesized, the sinking of oil-particle aggregates (“flocculent blizzard”) may have purged the water column from particles and DWH oil, leading to the deposition of natural and petroleum derived hydrocarbons to the seafloor.

Additionally, oil-particle aggregates in 2010–2011 may have formed with hydrocarbons different from crude or dissolved oil in the water column. We observed elevated concentrations of HMW PAHs at DSH10, the deepest site in 2011 (Table 2). Although HMW PAHs (pyrogenic) that are associated with atmospheric deposition and riverine inputs have been observed in sediment cores from other regions in the GoM [59,70] PAH source identification in deepwater sediments in the GoM are yet to be fully understood. PAH composition at DSH10 site does not indicate atmospheric deposition during 2010–2011 based on the ratio (BaP+BGP)/HMW (Table 3) used as an indicator for traffic-related pollution [80,81]. Therefore, there are three possible sources that can explain the elevated concentrations of HMW PAHs in 2011: 1) the large extent of the surface oil slick in 2010 was exposed to relatively high summer temperatures (25–30°C) [82] that enhanced the evaporation of the oil released [10]. Moderate evaporation of oil mousse was observed with preferential loss of LMW PAHs and <C14 *n*-alkanes [82] leaving a heavier residue (high in HMW PAHs) in the environment that formed oil-particle aggregates that sink. 2) Another possibility is deposition of burned products produced by *in situ* combustion of surface oil slicks during the summer of 2010 (6% of the leaked mass or 222,000–313,000 barrels) [10,83]. Burn residues are known to leave residues rich in HMW PAHs [40]. To our knowledge, there are no specific PAH ratios that identify burned residues from *in situ* combustion of DWH oil. 3) Diesel exhaust from thousands of vessels operating in the area during summer of 2010 (up to ~6000 vessels in July 2010) may have been a source of HMW PAHs to the aquatic environment based on the diagnostic ratio (ID+C)/HMW [80,81] observed at DSH10 site (Table 3).

Poor correlation between TOC fluxes and hydrocarbon concentrations, and differential PAH abundance between HMW and LMW compounds at PCB06 site located at 1043 m depth (Fig 2) suggest the sinking of oil-particle aggregates was not the only transport pathway in 2010. Another possible depositional mechanism is the advective transport and subsequent contact of the deep plume with the continental slope surface sediments (“bathtub ring” hypothesis). At PCB06 site, specific sediment intervals in 2010 showed a dominance of LMW over HMW PAHs (Fig 2). Typically in surface oil spills, LMW compounds are lost through rapid evaporation limiting its dissolution into the water column, contrary to observations in the DWH deep plume [10,14]. Insoluble non-volatile compounds formed surface slicks while more soluble compounds were entrained in the subsurface plume [10]. Therefore, the concentration of LMW PAHs is a promising utility to track plume-derived hydrocarbons to deep-sea sediments, contrary to only using one insoluble compound not abundant in the plume such as 17α(H)21β(H)-hopane to describe this transport pathway [84]. Because there was no observed increase in sediment deposition rates at the time LMW PAHs were higher in concentration in PCB06 (4–8 mm interval in 2010), a direct contact of the deep plume with the surface sediment might have occurred. This is supported by an oil droplet model that suggests the deep plume impinged on the continental slope in the DeSoto Canyon [85] near the two shallower sites in our study (PCB06, DSH08). In addition, it is possible that the two proposed mechanisms of hydrocarbon deposition can occur simultaneously. For example, DSH08 at 0–2 mm in 2010 presented increased concentration of LMW PAHs as well as TOC rates indicating a possible hydrocarbon input from sinking oil-particle aggregates and from the direct contact of the deep plume with the surface sediments (Fig 2).

## Conclusion

The analyses of DeSoto Canyon sediments show the contribution of the DWH event to sediment hydrocarbon content and composition, and provides insight into the transport pathways for hydrocarbons to the seafloor and their fate during 2010–2011. Higher hydrocarbon concentrations, and associated mixed hydrocarbon compositions from various sources (including biogenic *n*-alkanes, and petrogenic and pyrogenic PAHs) were observed during 2010–2011 in the DeSoto Canyon. Evidences were found for two hypothesized transport pathways of hydrocarbons: sinking of oil-particle aggregates and the direct contact of the deep plume with the sediment surface. Our results underline the complexity of hydrocarbons deposition due to weathering during transport, multiple sources, variable concentrations, and spatial (depth-related) variability in the DeSoto Canyon in 2010–2011. Further understanding of the interplay between the water column and sedimentary environments and the relative proportion of compounds deposited is critical for predicting the long-term fate of hydrocarbons from deep-water blowout events.

## Supporting Information

S1 Fig Geochemical profiles for aliphatics, 2–3 ring (low molecular weight, LMW) and 4–6 ring (high molecular weight, HMW) PAHs, and biomarkers (hopanes, steranes, disteranes) in the control site, NT1200. (TIF)

S1 Table Short-lived radioisotope (^210^Pb, ^234^Th) activities and constant rate of supply age model for the cores collected in 2010. (DOCX)

## References

[1] Gordon ES, Goñi Ma (2004) Controls on the distribution and accumulation of terrigenous organic matter in sediments from the Mississippi and Atchafalaya river margin. Mar Chem 92: 331–352. 10.1016/j.marchem.2004.06.035

[2] Waterson EJ, Canuel EA (2008) Sources of sedimentary organic matter in the Mississippi River and adjacent Gulf of Mexico as revealed by lipid biomarker and 13CTOC analyses. Org Geochem 39: 422–439.

[3] Goni MA, Ruttenberg K, Eglinton TI (1998) A reassessment of the sources and importance of land-derived organic matter in surface sediments from the Gulf of Mexico. Geochim Cosmochim Acta 62: 3055–3075.

[4] Gordon ES, Goñi Ma (2003) Sources and distribution of terrigenous organic matter delivered by the Atchafalaya River to sediments in the northern Gulf of Mexico. Geochim Cosmochim Acta 67: 2359–2375. 10.1016/S0016-7037(02)01412-6

[5] Song Z, Wang L, Liu J, Wang C, Chen D (2008) Characterizing organic matter in marine sediments associated with gas hydrate and oil seepage from the Gulf of Mexico. Geofluids 8: 293–300. 10.1111/j.1468-8123.2008.00225.x

[6] Kennicutt MC II, Brooks JM, Atlas EL, Giam CS (1988) Organic compounds of environmental concern in the Gulf of Mexico " a review. Aquat Toxicol 11: 191–212.

[7] Ocean Studies Board and Marine Board (2003) Oil in the Sea III. Inputs, fates, and effects National Academies Press, Washington, D.C. 25057607

[8] McNutt MK, Chu S, Lubchenco J, Hunter T, Dreyfus G, Murawski SA, et al (2012) Applications of science and engineering to quantify and control the Deepwater Horizon oil spill. Proc Natl Acad Sci U S A 109: 20222–20228. 10.1073/pnas.1214389109 23213225 PMC3528582

[9] Murawski SA, Hogarth WT, Peebles EB, Barbeiri L (2014) Prevalence of External Skin Lesions and Polycyclic Aromatic Hydrocarbon Concentrations in Gulf of Mexico Fishes, Post-Deepwater Horizon. Trans Am Fish Soc 143: 37–41. 10.1080/00028487.2014.911205

[10] Ryerson TB, Camilli R, Kessler JD, Kujawinski EB, Reddy CM, Valentine DL, et al (2012) Chemical data quantify Deepwater Horizon hydrocarbon flow rate and environmental distribution. Proc Natl Acad Sci U S A 109: 20246–20253. 10.1073/pnas.1110564109 22233807 PMC3528560

[11] Camilli R, Reddy CM, Yoerger DR, Van Mooy BAS, Jakuba M V., Kinsey JC, et al (2010) Tracking Hydrocarbon Plume Transport and Biodegradation at Deepwater Horizon. Science (80-) 330: 201–204. 20724584 10.1126/science.1195223

[12] Diercks A-R, Highsmith RC, Asper VL, Joung D, Zhou Z, Guo L, et al (2010) Characterization of subsurface polycyclic aromatic hydrocarbons at the Deepwater Horizon site. Geophys Res Lett 37: 1–6. 10.1029/2010GL045046

[13] Joye SB, MacDonald IR, Leifer I, Asper V (2011) Magnitude and oxidation potential of hydrocarbon gases released from the BP oil well blowout. Nat Geosci 4: 160–164. 10.1038/ngeo1067

[14] Reddy CM, Arey JS, Seewald JS, Sylva SP, Lemkau KL, Nelson RK, et al (2011) Composition and fate of gas and oil released to the water column during the Deepwater Horizon oil spill. Proc Natl Acad Sci U S A 109: 20229–20234. 10.1073/pnas.1101242108 21768331 PMC3528605

[15] Valentine DL, Kessler JD, Redmond MC, Mendes SD, Heintz MB, Farwell C, et al (2010) Propane respiration jump-starts microbial response to a deep oil spill. Science (80-) 330: 208–211. 10.1126/science.1196830 20847236

[16] Passow U, Ziervogel K, Asper V, Diercks A (2012) Marine snow formation in the aftermath of the Deepwater Horizon oil spill in the Gulf of Mexico. Environ Res Lett 7: 035301 10.1088/1748-9326/7/3/035301

[17] Ziervogel K, McKay L, Rhodes B, Osburn CL, Dickson-Brown J, Arnosti C, et al (2012) Microbial activities and dissolved organic matter dynamics in oil-contaminated surface seawater from the Deepwater Horizon oil spill site. PLoS One 7: e34816 10.1371/journal.pone.0034816 22509359 PMC3324544

[18] Graham WM, Condon RH, Carmichael RH, D’Ambra I, Patterson HK, Linn LJ, et al (2010) Oil carbon entered the coastal planktonic food web during the Deepwater Horizon oil spill. Environ Res Lett 5: 045301 10.1088/1748-9326/5/4/045301

[19] Mitra S, Kimmel DG, Snyder J, Scalise K, Mcglaughon BD, Roman MR, et al (2012) Macondo-1 well oil-derived polycyclic aromatic hydrocarbons in mesozooplankton from the northern Gulf of Mexico. Geophys Res Lett 39: 1–7. 10.1029/2011GL049505

[20] Kessler JD, Valentine DL, Redmond MC, Du M, Chan EW, Mendes SD, et al (2011) A persistent oxygen anomaly reveals the fate of spilled methane in the deep Gulf of Mexico. Science 331: 312–315. 10.1126/science.1199697 21212320

[21] Kostka JE, Prakash O, Overholt W a, Green SJ, Freyer G, Canion A, et al (2011) Hydrocarbon-degrading bacteria and the bacterial community response in gulf of Mexico beach sands impacted by the deepwater horizon oil spill. Appl Environ Microbiol 77: 7962–7974. 10.1128/AEM.05402-11 21948834 PMC3208977

[22] Whitehead A, Dubansky B, Bodinier C, Garcia TI, Miles S, Pilley C (2011) Genomic and physiological footprint of the Deepwater Horizon oil spill on resident marsh fishes: 1–5. 10.1073/pnas.1109545108 PMC3528528 21949382

[23] Chanton JP, Cherrier J, Wilson RM, Sarkodee-Adoo J, Bosman S, Mickle A, et al (2012) Radiocarbon evidence that carbon from the Deepwater Horizon spill entered the planktonic food web of the Gulf of Mexico. Environ Res Lett 7: 045303 10.1088/1748-9326/7/4/045303

[24] Lin Q, Mendelssohn IA (2012) Impacts and Recovery of the Deepwater Horizon Oil Spill on Vegetation Structure and Function of Coastal Salt Marshes in the Northern Gulf of Mexico. October. 10.1021/es203552p 22369124

[25] Valentine DL, Mezic I, Mac S, Fonoberov VA, Loire S (2011) Dynamic autoinoculation and the microbial ecology of a deep water hydrocarbon irruption. PNAS. 10.1073/pnas.1108820109 PMC3528554 22233808

[26] Valentine MM, Benfield MC (2013) Characterization of epibenthic and demersal megafauna at Mississippi Canyon 252 shortly after the Deepwater Horizon Oil Spill. Mar Pollut Bull 77: 196–209. 10.1016/j.marpolbul.2013.10.004 24269011

[27] Montagna P a, Baguley JG, Cooksey C, Hartwell I, Hyde LJ, Hyland JL, et al (2013) Deep-sea benthic footprint of the deepwater horizon blowout. PLoS One 8: e70540 10.1371/journal.pone.0070540 23950956 PMC3737147

[28] White HK, Hsing P-Y, Cho W, Shank TM, Cordes EE, Quattrini AM, et al. (2012) Impact of the Deepwater Horizon oil spill on a deep-water coral community in the Gulf of Mexico. PNAs. 10.1073/pnas.1118029109 PMC3528508 22454495

[29] Chang Y-L, Oey L, Xu F-H, Lu H-F, Fujisaki A (2011) 2010 Oil Spill: Trajectory Projections Based on Ensemble Drifter Analyses. Ocean Dyn 61: 829–839. 10.1007/s10236-011-0397-4

[30] Weisberg RH, Zheng L, Liu Y (2011) Tracking Subsurface Oil in the Aftermath of the Deepwater Horizon Well Blowout. Geophys Monogr Ser 195: 205–215.

[31] Engstrom DR (1993) A lightweight extruder for accurate sectioning of soft-bottom lake sediment cores in the field. Limnol Oceanogr 38: 1796–1802.

[32] Valsangkar AB (2007) A device for finer-scale sub-sectioning of aqueous sediments. Curr Sci 92: 5–8.

[33] Appleby PG, Oldfield F (1978) The assessment of 210 Pb data from sites with varying sediment accumulation rates. Hydrobiologia 103: 29–35.

[34] Binford MW (1990) Calculation and uncertainty analysis of 210Pb dates for PIRLA project lake sediment cores. J Paleolimnol 3: 253–267.

[35] Schwing P, Romero I, Brooks G, Hastings D, Larson R, Hollander D (2015) A Decline in Benthic Foraminifera Following the Deepwater Horizon Event in the Northeastern Gulf of Mexico. PLoS One. 10.1371/journal.pone.0120565 PMC4364910 25785988

[36] Brodie CR, Casford JSL, Lloyd JM, Leng MJ, Heaton THE, Kendrick CP, et al (2011) Evidence for bias in C/N, δ13C and δ15N values of bulk organic matter, and on environmental interpretation, from a lake sedimentary sequence by pre-analysis acid treatment methods. Quat Sci Rev 30: 3076–3087. 10.1016/j.quascirev.2011.07.003

[37] 8270D EM (2007) Semivolatile Organic Compounds by Gas Chromatography/Mass Spectrometry (Gc/Ms): 1–72.

[38] 8015C EM (2007) Nonhalogenated Organics by Gas Chromatography: 1–68.

[39] Dickhut RM, Canuel E a., Gustafson KE, Liu K, Arzayus KM, Walker SE, et al (2000) Automotive Sources of Carcinogenic Polycyclic Aromatic Hydrocarbons Associated with Particulate Matter in the Chesapeake Bay Region. Environ Sci Technol 34: 4635–4640. 10.1021/es000971e

[40] Wang Z, Fingas MF, Landriault M, Sigouin L, Lambert P, Turpin R, et al (1999) PAH Distribution in the 1994 and 1997 Mobile Burn Products and Determination of the Diesel PAH Destruction Efficiencies. Int Oil Spill Conf Proc 1999: 1287–1292. 10.7901/2169-3358-1999-1-1287

[41] Yunker MB, Macdonald RW (2003) Alkane and PAH depositional history, sources and fluxes in sediments from the Fraser River Basin and Strait of Georgia, Canada. Org Geochem 34: 1429–1454. 10.1016/S0146-6380(03)00136-0

[42] Zhang Z, Huang J, Yu G, Hong H (2004) Occurrence of PAHs, PCBs and organochlorine pesticides in the Tonghui River of Beijing, China. Environ Pollut 130: 249–261. 10.1016/j.envpol.2003.12.002 15158038

[43] Li G, Xia X, Yang Z, Wang R, Voulvoulis N (2006) Distribution and sources of polycyclic aromatic hydrocarbons in the middle and lower reaches of the Yellow River, China. Environ Pollut 144: 985–993. 10.1016/j.envpol.2006.01.047 16603293

[44] Wang Z, Fingas MF (2003) Development of oil hydrocarbon fingerprinting and identification techniques. Mar Pollut Bull 47: 423–452. 10.1016/S0025-326X(03)00215-7 12899888

[45] Aeppli C, Nelson RK, Radović JR, Carmichael C a, Valentine DL, Reddy CM. (2014) Recalcitrance and degradation of petroleum biomarkers upon abiotic and biotic natural weathering of Deepwater Horizon oil. Environ Sci Technol 48: 6726–6734. 10.1021/es500825q 24831878

[46] Mulabagal V, Yin F, John GF, Hayworth JS, Clement TP (2013) Chemical fingerprinting of petroleum biomarkers in Deepwater Horizon oil spill samples collected from Alabama shoreline. Mar Pollut Bull 70: 147–154. 10.1016/j.marpolbul.2013.02.026 23523118

[47] Operational Science Advisory Team (OSAT 1) (2010) Summary Report for Sub-Sea and Sub-Surface Oil and Dispersant Detection: Sampling and Monitoring.

[48] Scarlett A, Galloway TS, Rowland SJ (2007) Chronic Toxicity of Unresolved Complex Mixtures (UCM) of Hydrocarbons in Marine Sediments. J soil sediments 7: 200–206.

[49] Hu L, Guo Z, Feng J, Yang Z, Fang M (2009) Distributions and sources of bulk organic matter and aliphatic hydrocarbons in surface sediments of the Bohai Sea, China. Mar Chem 113: 197–211. 10.1016/j.marchem.2009.02.001

[50] Ahad JME, Ganeshram RS, Bryant CL, Cisneros-dozal LM, Ascough PL, Fallick AE, et al (2011) Sources of n-alkanes in an urbanized estuary: Insights from molecular distributions and compound-specific stable and radiocarbon isotopes. Mar Chem 126: 239–249. 10.1016/j.marchem.2011.06.002

[51] Nemr A El, El-Sadaawy MM, Khaled A, Draz SO (2012) Aliphatic and polycyclic aromatic hydrocarbons in the surface sediments of the Mediterranean: assessment and source recognition of petroleum hydrocarbons. Environ Monit Assess. 10.1007/s10661-012-2889-1 23054267

[52] Wang C, Chen B, Zhang B, He S, Zhao M (2013) Fingerprint and weathering characteristics of crude oils after Dalian oil spill, China. Mar Pollut Bull. 10.1016/j.marpolbul.2013.03.034 23623662

[53] Yu CC, Zeng EY (1992) Petrogenic and Biogenic Sources of N-Alkanes off San Diego, California: 53–62.

[54] Barakat AO, Mostafa AR, Qian Y, Kennicutt MC II (2002) Application of Petroleum Hydrocarbon Aplication of Petroleum Hydrocarbon Chemical Fingerprinting in Oil Spill Investigations——Gulf of Suez, Egypt. Spill Sci Technol Bull 7: 229–239.

[55] Barakat AO, Mostafa A, Wade TL, Sweet ST, El Sayed NB (2011) Distribution and characteristics of PAHs in sediments from the Mediterranean coastal environment of Egypt. Mar Pollut Bull 62: 1969–1978. 10.1016/j.marpolbul.2011.06.024 21764083

[56] Tansel B, Fuentes C, Sanchez M, Predoi K, Acevedo M (2011) Persistence profile of polyaromatic hydrocarbons in shallow and deep Gulf waters and sediments: effect of water temperature and sediment-water partitioning characteristics. Mar Pollut Bull 62: 2659–2665. 10.1016/j.marpolbul.2011.09.026 22018883

[57] McGroddy SE, Farrington JW (1995) Sediment porewater partitioning of polycyclic aromatic hydrocarbons in three cores from Boston harbor, massachusetts. Environ Sci Technol 29: 1542–1550. 10.1021/es00006a016 22276875

[58] Rowe GT, Kennicutt II MC (2009) Northern Gulf of Mexico Continental Slope Habitats and Benthic Ecology Study: Final Report. US. Dept. of the Interior, Minerals Management. Service, Gulf of Mexico OCS Region, New Orleans, LA. OCS Study MMS 2009–039. 456 pp.

[59] Presley BJ, Wade TL, Santschi P, Baskaran M (1998) Historical Contamination of Mississippi River Delta, Tampa Bay, and Galveston Bay Sediments US Department of Commerce. Silver Springs, Maryland.

[60] Santschi PH, Presley BJ, Wade TL, Garcia-Romero B, Baskaran M (2001) Historical contamination of PAHs, PCBs, DDTs, and heavy metals in Mississippi River Delta, Galveston Bay and Tampa Bay sediment cores. Mar Environ Res 52: 51–79. 11488356 10.1016/s0141-1136(00)00260-9

[61] Qiao M, Wang C, Huang S, Wang D, Wang Z (2006) Composition, sources, and potential toxicological significance of PAHs in the surface sediments of the Meiliang Bay, Taihu Lake, China. Environ Int 32: 28–33. 10.1016/j.envint.2005.04.005 15996733

[62] El Nemr A, El-Sadaawy MM, Khaled A, Draz SO (2012) Aliphatic and polycyclic aromatic hydrocarbons in the surface sediments of the Mediterranean: assessment and source recognition of petroleum hydrocarbons. Environ Monit Assess 185: 4571–4589. 10.1007/s10661-012-2889-1 23054267

[63] Baumard P, Budzinski H, Garrigues P, Sorbe JC, Burgeot T, Bellocq J (1998) Concentrations of PAHs (polycyclic aromatic hydrocarbons) in various marine organisms in relation to those in sediments and to trophic level. Mar Pollut Bull 36: 951–960. 10.1016/S0025-326X(98)00088-5

[64] Commendatore MG, Nievas ML, Amin O, Esteves JL (2012) Sources and distribution of aliphatic and polyaromatic hydrocarbons in coastal sediments from the Ushuaia Bay (Tierra del Fuego, Patagonia, Argentina). Mar Environ Res 74: 20–31. 10.1016/j.marenvres.2011.11.010 22189069

[65] Long ER, Macdonald DD, Smith SL, Calder FD (1995) Incidence of Adverse Biological Effects Within Ranges of Chemical Concentrations in Marine and Estuarine Sediments. Environ Manage 19: 81–97.

[66] Schneider a R, Stapleton HM, Cornwell J, Baker JE (2001) Recent declines in PAH, PCB, and toxaphene levels in the northern Great Lakes as determined from high resolution sediment cores. Environ Sci Technol 35: 3809–3815. 11642437 10.1021/es002044d

[67] Goni MA, Rutternberg KC, Eglinton TI (1997) Sources and contribution of terrigenous organic carbon to surface sediments in the Gulf of Mexico. Lett to Nat 389: 275–278.

[68] Reide Corbett D, McKee B, Allison M (2006) Nature of decadal-scale sediment accumulation on the western shelf of the Mississippi River delta. Cont Shelf Res 26: 2125–2140. 10.1016/j.csr.2006.07.012

[69] Kujau A, Nürnberg D, Zielhofer C, Bahr A, Röhl U (2010) Mississippi River discharge over the last ~560,000years—Indications from X-ray fluorescence core-scanning. Palaeogeogr Palaeoclimatol Palaeoecol 298: 311–318. 10.1016/j.palaeo.2010.10.005

[70] Mitra S, Bianchi T. (2003) A preliminary assessment of polycyclic aromatic hydrocarbon distributions in the lower Mississippi River and Gulf of Mexico. Mar Chem 82: 273–288. 10.1016/S0304-4203(03)00074-4

[71] Peterson CH, Kennicutt MC II, Green RH, Montagna P, Harper J. DE, Powell EN, et al (1996) Ecological consequences of environmental perturbations associated with offshore hydrocarbon production: a perspective on long-term exposures in the Gulf of Mexico. Can J Fish Aquat Sci 53: 2637–2654. 10.1139/cjfas-53-11-2637

[72] Macdonald IR, Leifer I, Sassen R, Stine P, Mitchell R, Guinasso N, et al. (2002) Transfer of hydrocarbons from natural seeps to the water column and atmosphere: 95–107.

[73] Iqbal J, Portier RJ, Gisclair D (2007) Aspects of petrochemical pollution in coastal Louisiana, USA. Mar Pollut Bull 54: 792–797. 17395213 10.1016/j.marpolbul.2007.02.004

[74] Wang C, Sun H, Chang Y, Song Z, Qin X (2011) PAHs distribution in sediments associated with gas hydrate and oil seepage from the Gulf of Mexico. Mar Pollut Bull 62: 2714–2723. 10.1016/j.marpolbul.2011.09.016 21982427

[75] Ruttenberg KC, Goni MA (1997) Phosphorus distribution, C:N:P ratios, and δ13C, in arctic, temperate, and tropical coastal sediments: tools for characterizing bulk sedimentary organic matter. 139: 123–145.

[76] Rosenheim BE, Pendergraft M a., Flowers GC, Carney R, Sericano JL, Amer RM, et al. (2014) Employing extant stable carbon isotope data in Gulf of Mexico sedimentary organic matter for oil spill studies. Deep Sea Res Part II Top Stud Oceanogr. 10.1016/j.dsr2.2014.03.020

[77] Hazen TC, Dubinsky E a, DeSantis TZ, Andersen GL, Piceno YM, Singh N, et al (2010) Deep-sea oil plume enriches indigenous oil-degrading bacteria. Science 330: 204–208. 10.1126/science.1195979 20736401

[78] Payne JR, Clayton JR, Kirstein BE (2003) Oil/Suspended Particulate Material Interactions and Sedimentation. Spill Sci Technol Bull 8: 201–221. 10.1016/S1353-2561(03)00048-3

[79] Khelifa A, Hill PS, Lee K (2005) The role of oil-sediment aggregation in dispersion and biodegradation of spilled oil In: Al-Azab M, El-Shorbagy W, Al-Ghais S, editors. Oil Pollution and its Environmental Impact in the Arabian Gulf Region. Elsevier B.V. pp. 131–145.

[80] Harrison RM, Smith DJT, Luhana L (1996) Source Apportionment of Atmospheric Polycyclic Aromatic Hydrocarbons Collected from an Urban Location in Birmingham, U.K. Environ Sci Technol 30: 825–832. 10.1021/es950252d

[81] Lang Y-H, Yang W (2014) Source apportionment of PAHs using Unmix model for Yantai costal surface sediments, China. Bull Environ Contam Toxicol 92: 30–35. 10.1007/s00128-013-1164-7 24292847

[82] Liu Z, Liu J, Zhu Q, Wu W (2012) The weathering of oil after the Deepwater Horizon oil spill: insights from the chemical composition of the oil from the sea surface, salt marshes and sediments. Environ Res Lett 7: 035302 10.1088/1748-9326/7/3/035302

[83] Schaum J, Cohen M, Perry S, Artz R, Draxler R, Frithsen JB, et al (2010) Screening level assessment of risks due to dioxin emissions from burning oil from the BP Deepwater Horizon Gulf of Mexico spill. Environ Sci Technol 44: 9383–9389. 10.1021/es103559w 21073188

[84] Valentine DL, Fisher GB, Bagby SC, Nelson RK, Reddy CM, et al. (2014) Fallout plume of submerged oil from Deepwater Horizon. Proc Natl Acad Sci. 10.1073/pnas.1414873111 PMC4234598 25349409

[85] Paris CB, Le M, Aman ZM, Subramaniam A, Helgers J, Wang D, et al (2012) Evolution of the Macondo Well Blowout: Simulating the Effects of the Circulation and Synthetic Dispersants on the Subsea Oil Transport. Environ Sci Technol 46: 13293–13302. 10.1021/es303197h 23167517

