# Supplementary figures and images for: Hydrocarbons in Deep-Sea Sediments following the 2010 Deepwater Horizon Blowout in the Northeast Gulf of Mexico
Source: PLoS One. 2015 May 28;10(5):e0128371. doi: 10.1371/journal.pone.0128371 (PMC4447447; doi:10.1371/journal.pone.0128371)

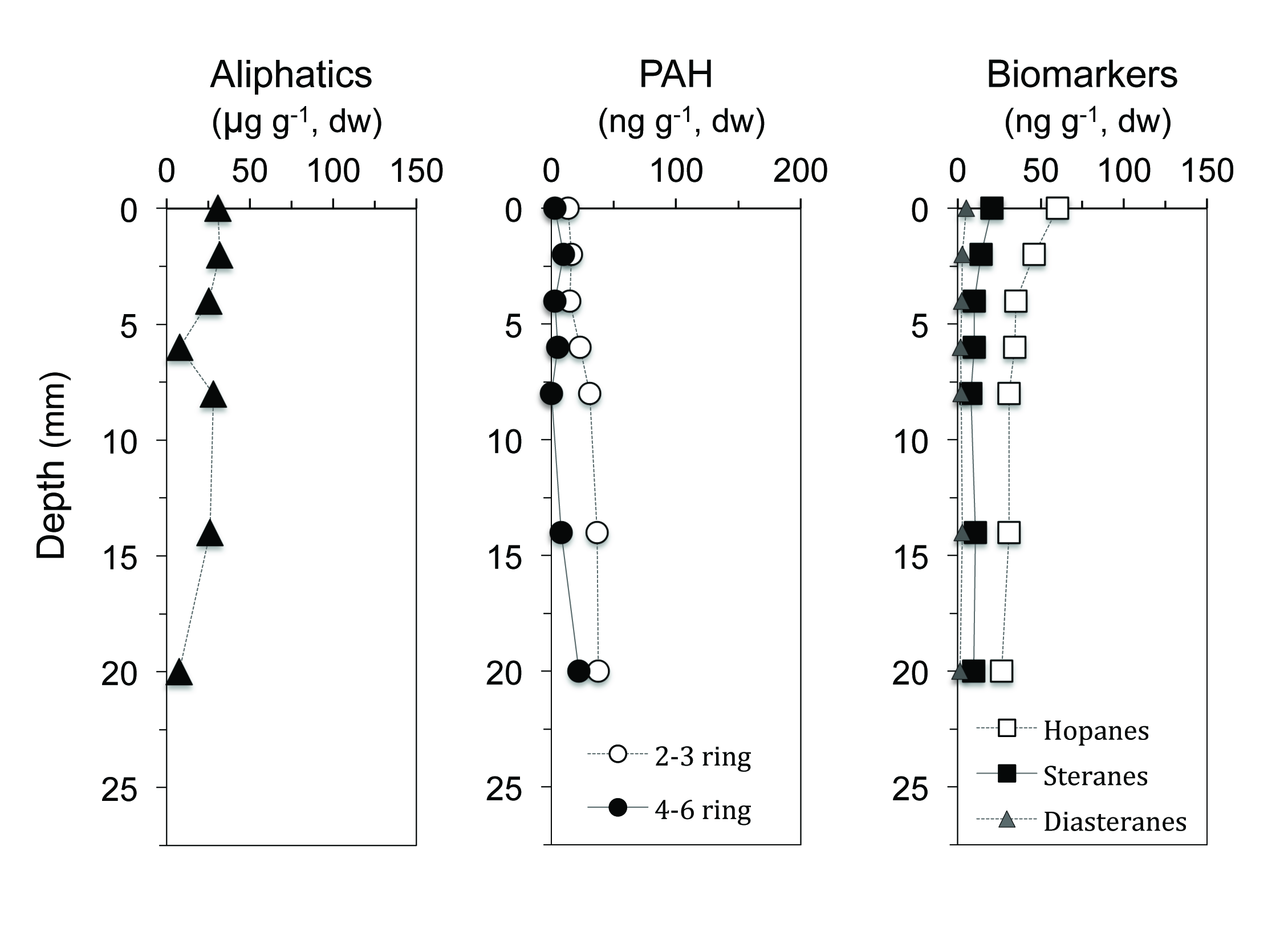

Supplement: S1 Fig — (TIF) [file pone.0128371.s001.tif]
