# Supplementary material for: Hydrocarbons in Deep-Sea Sediments following the 2010 Deepwater Horizon Blowout in the Northeast Gulf of Mexico
Source: PLoS One. 2015 May 28;10(5):e0128371. doi: 10.1371/journal.pone.0128371 (PMC4447447; doi:10.1371/journal.pone.0128371)
Supplement: S1 Table — (DOCX) [file pone.0128371.s002.docx]

**Table S1.** Short-lived radioisotope (^210^Pb, ^234^Th) activities and constant rate of supply age model for the cores collected in 2010.
